# Supplementary material for: 3D bulk-resolved g-wave altermagnetic order parameter in CrSb
Source: Nature. 2026 Aug 26;656(8129):854–60. doi: 10.1038/s41586-026-10902-z (PMC13518244; doi:10.1038/s41586-026-10902-z)
Supplement: Supplementary file 1 [file 41586_2026_10902_MOESM1_ESM.pdf]

nature portfolio

https://doi.org/10.1038/s41586-026-10902-z

# Supplementary information

# 3D bulk-resolved *g*-wave altermagnetic order parameter in CrSb

In the format provided by the
authors and unedited

# Supplementary information for:

## 3D bulk-resolved $g$-wave altermagnetic order parameter in CrSb

Mengmeng Long, Theodore I. Weinberger, Zheyu Wu,
Mads F. Hansen, Ran Tao, Mridul Shrestha, Dave Graf,
Yurii Skourski, F. Malte Grosche, Alexander G. Eaton

## Contents

**1 Correspondence Between Theoretically-Predicted Fermi Surface and Quantum Oscillation Data 2**

1.1 Choice of Exchange Correlation Functional and Double Counting . . . . . . . 3

1.2 Effect of Correlations on the Topology of the Fermi Surface . . . . . . . . . . 4

1.3 Effect of Spin-Orbit Coupling . . . . . . . . . . . . . . . . . . . . . . . . . . . 6

1.4 Magnetic Breakdown of the Fermi Surface . . . . . . . . . . . . . . . . . . . . . . 8

1.5 Simulated Orbits for Field Along $ab$ Direction . . . . . . . . . . . . . . . . . . . 15

**2 Summary 22**

# 1 Correspondence Between Theoretically-Predicted Fermi Surface and Quantum Oscillation Data

A definitive identification of unconventional magnetic phases relies on the precise momentum-space mapping of nodal planes associated with their magnetic order parameters. As established in the main text, de Haas-van Alphen quantum oscillations (QOs) constitute a powerful, bulk-sensitive technique that can provide a 3D map of symmetry breaking induced in the electronic structure at the onset of magnetism. By resolving trends in the $k$-space cross-sectional area of quasiparticle orbits, QO measurements are capable of directly resolving the location of any nodal planes associated with the momentum-dependent spin splitting of the Fermi surface inherent to metallic altermagnets. However, establishing an unambiguous correspondence between idealised density functional theory (DFT) predictions and experimental QO frequency spectra requires a complete Fermi surface determination that accounts for the complex interplay of electronic correlations, spin-orbit coupling (SOC), and high-field quasiparticle dynamics.

This Supplementary Information details a framework for mapping QO measurements in altermagnets to DFT calculations, which we apply to our experimental observations in CrSb. We systematically incorporate many-body correlations to accurately reproduce the experimental Fermi-surface topology, thereby physically justifying a Lifshitz transition induced by shifting the band edges imposed in the main text that closes the central sheets of the Fermi surface. Furthermore, while collinear altermagnetism enforces strict band degeneracies along nodal planes, the introduction of SOC inherently lifts these degeneracies, inducing hybridisation gaps that formally alter the low-energy orbital contours. By quantitatively evaluating these SOC-induced gaps against the cyclotron energy, we demonstrate that magnetic breakdown readily occurs under the strong magnetic fields utilised in our measurements. This tunnelling effectively restores the unhybridised altermagnetic orbits, providing the basis to extract the $g$-wave order parameter

of CrSb from the angular dependence of observed QO frequencies.

### 1.1 Choice of Exchange Correlation Functional and Double Counting

To accurately capture the electronic correlations and ground-state magnetic properties of CrSb, we systematically compared different choices of exchange-correlation functional alongside the double-counting scheme within the DFT+$U$ framework as employed within `WIEN2k`.[1] We compared the predicted moment against the experimentally determined local magnetic moment of CrSb, which neutron diffraction results establish to be approximately 2.7 $\mu_{\mathrm{B}}$ per chromium atom.[2]

At the baseline DFT level ($U = 0$ eV), the Generalised Gradient Approximation (GGA) yields a local magnetic moment of 2.76 $\mu_{\mathrm{B}}$, slightly higher than the Local Density Approximation (LDA) value of 2.61 $\mu_{\mathrm{B}}$, which is expected due to the tendency of GGA functionals to further localise $d$-electron states. The inclusion of a Hubbard $U$ parameter significantly modifies these moments depending on the applied double-counting correction (Fig. S1). Utilising the Self-Interaction Correction (SIC) scheme with GGA monotonically increases the local moment far beyond the experimentally determined value, reaching 3.58 $\mu_{\mathrm{B}}$ at $U = 4$ eV. This is the expected behaviour of SIC counting because it penalises partial occupancies, enhancing exchange splitting, and increasing the onsite moment.[3] In contrast, the Around Mean Field (AMF) scheme assumes a more uniform baseline correlation, mitigating this over-localisation. While the GGA (AMF) configuration proves unstable, with the moment collapsing to 1.80 $\mu_{\mathrm{B}}$ at $U = 4$ eV, the LDA (AMF) approach remains remarkably robust up to $U = 4$ eV.[4] Consequently, we selected the LDA functional coupled with AMF double counting to study the evolution of the topology of the Fermi surface as a function of the Hubbard $U$ parameter. We note that the choice of AMF counting is perhaps unsurprising since CrSb is metallic and only moderately correlated.

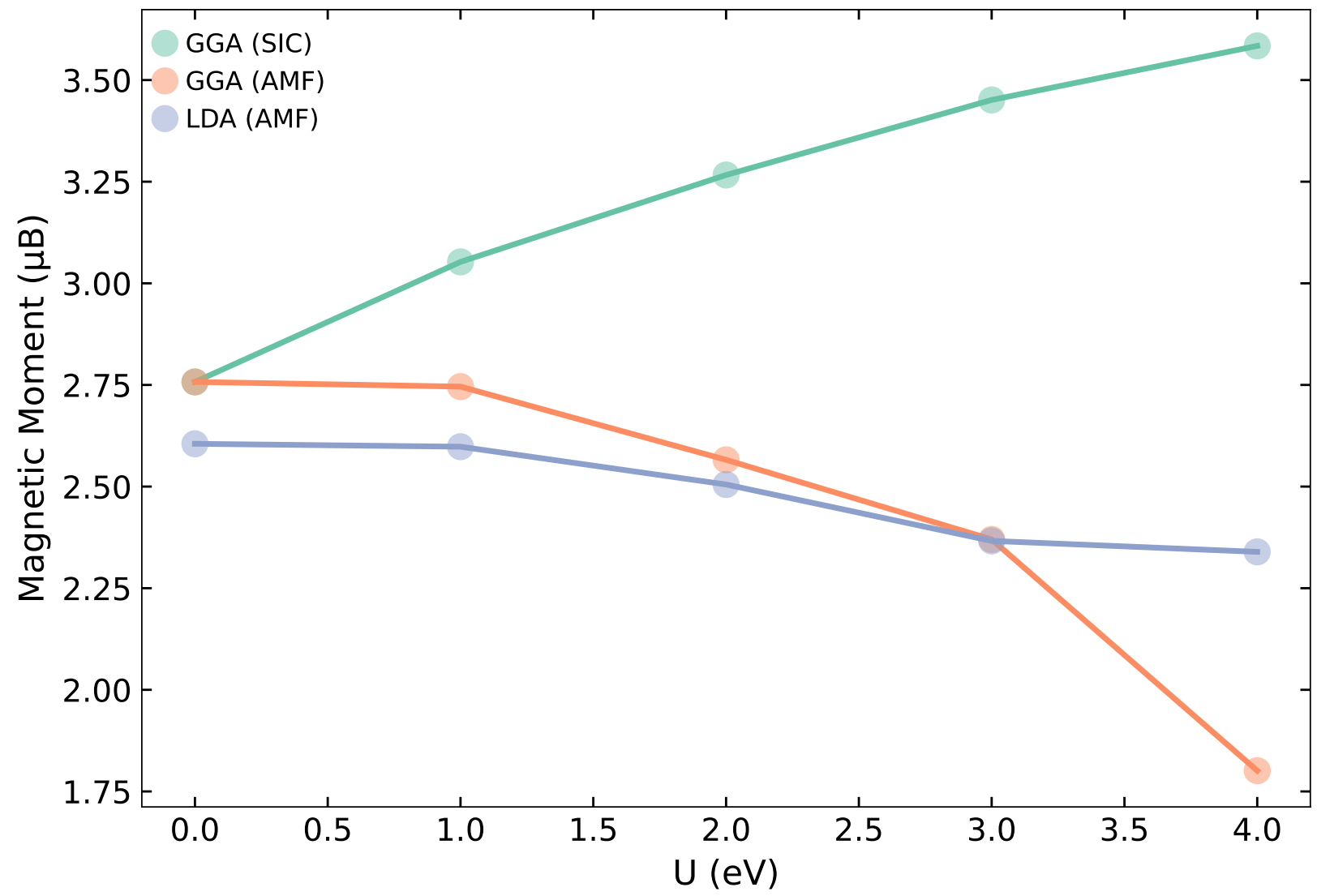


**Fig. S1. Calculated magnetic moment per chromium site as a function of the Hubbard $U$ parameter using different exchange correlation functionals and double counting schema.** At $U = 0$ eV, all calculational methods predict a moment of $\approx 2.7\ \mu_B$, in good agreement with the measured value. The GGA-based calculations with SIC double counting exhibit a steadily growing magnetic moment with increasing $U$, reaching $\approx 3.5\ \mu_B$ at $U = 4$ eV. GGA with AMF yields a magnetic moment that collapses at high $U$. The LDA (AMF) scheme yields a stable magnetic moment that remains consistent with the experimentally determined value of approximately $2.7\ \mu_B$ across the range of $U$ values investigated.

## 1.2 Effect of Correlations on the Topology of the Fermi Surface

Applying a Hubbard $U$ correction to the correlated chromium $d$-orbitals increases the energy penalty for double occupancy, thereby shifting these states to higher energies relative to the Fermi level. This correlation effect qualitatively provides physical motivation for the band-edge shift applied in the main text. Consequently, as the correlation strength increases, the open cylindrical Fermi surface sheet surrounding the Γ point – as predicted by vanilla DFT ($U = 0$ eV) – is expected to undergo a Lifshitz transition (Fig. S2). Indeed, our LDA+$U$ calculations reveal that while the sheet remains open for $U \leq 2.5$ eV, a Hubbard parameter of $U > 2.5$ eV is sufficient to close the ends of this central sheet near the Brillouin zone boundary.

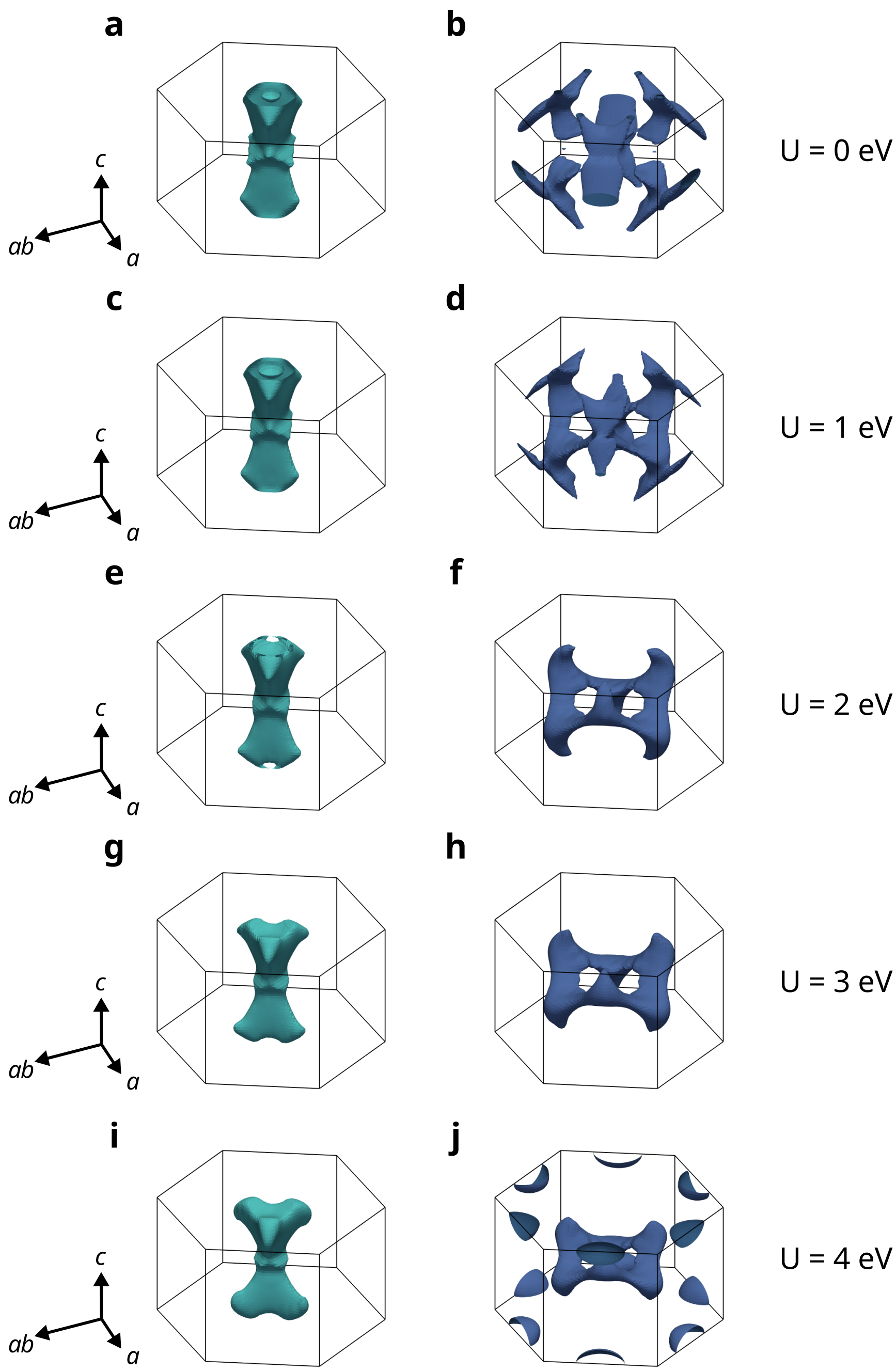


**Fig. S2. Correlation-induced Lifshitz transition of the electron Fermi surface.** (a,b) Standard DFT calculations ($U = 0$ eV) predict an open cylindrical topology for the central electron sheet. Increasing the Hubbard interaction through (c,d) $U = 1$ eV and (e,f) $U = 2$ eV progressively pinches the sheet at the Brillouin zone boundaries. A Lifshitz transition occurs between 2 and 3 eV, resulting in the formation of the closed, isolated 'dogbone' pocket observed at (g,h) $U = 3$ eV and (i,j) $U = 4$ eV, which matches the experimental observation. Fermi surfaces plotted using `py_FS`.[5,6]

This correlation-induced Lifshitz transition naturally yields the closed 'dogbone' topology, reconciling our DFT calculations with the QO measurements without the need for an ad hoc Fermi level adjustment.

## 1.3 Effect of Spin-Orbit Coupling

The concept of a 'pristine' altermagnet where spin-up and spin-down Fermi surface sheets remain strictly degenerate along symmetry-enforced nodal planes[7,8] is a theoretical idealisation. In reality, some degree of SOC will generally be present in a crystalline solid. Therefore, even a minimal SOC interaction will mix the spin-up and spin-down channels, lifting degeneracies along the nodal plane in physical materials. This interaction can cause previously intersecting Fermi sheets to hybridise and open finite energy/momentum gaps.

Consequently, understanding the effect of SOC is critical when interpreting QO data in altermagnets.[9] The magnitude of the SOC-induced gaps must be well understood in relation to the energy scale of the cyclotron orbits to determine whether a quasiparticle will traverse the hybridised band structure, or see an 'effective' spin-split Fermi surface.

We constructed a minimal tight-binding model to provide precise parametric control over the SOC interaction. We began by performing a Wannier decomposition of the non-relativistic DFT band structure.[10–12] We chose our $U = 3.5$ eV calculation for this, where $U$ should be sufficiently large to stabilise the closing of the 'dogbone' sheets. The relevant low-energy states were projected onto a basis consisting of five $3d$-orbitals for each of the two chromium atoms and three $5p$-orbitals for each of the two antimony atoms in the primitive unit cell, which are the dominant orbital contributions about the Fermi level (see Fig. S3). Including the spin degree of freedom ($|\uparrow\rangle, |\downarrow\rangle$), this decomposition yields a $32 \times 32$ Hamiltonian, $\mathcal{H}_0$. Our Wannier decomposition was then symmetrised according to the point group operations of the magnetic unit cell.[13] To ensure a robust yet computationally tractable model, the real-space hopping

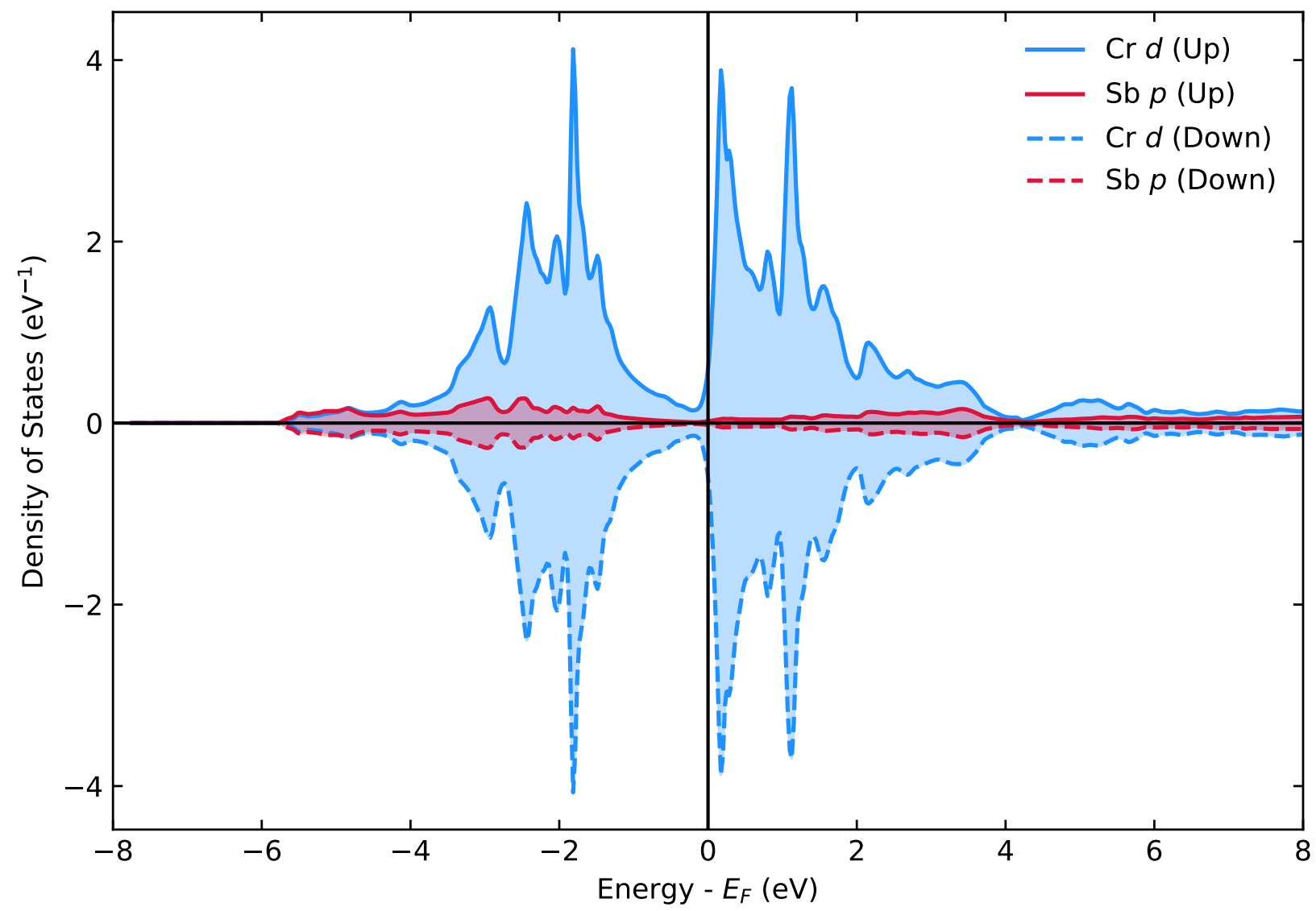


**Fig. S3. Projected density of states (PDOS) onto the antimony $p$-orbitals and chromium $d$-orbitals of CrSb.** The electronic structure near the Fermi level ($E_F$ = 0 eV) is primarily dominated by chromium $3d$-states (light blue), with a significantly smaller contribution from antimony $5p$-orbitals (red). The spin-up (solid lines) and spin-down (dashed lines) channels are shown on the positive and negative axes, respectively. This dominance of light transition metal $d$-states suggests that the contribution of SOC at the Fermi surface should be small, inducing hybridisation but not substantial reconstruction.

parameters were truncated to retain only those with a maximum $\mathbf{R}$-vector of $(2, 2, 2)$ and an energy magnitude exceeding $10^{-3}$ eV on a $k$-space mesh of $(120, 120, 60)$ for QO calculations, and an $\mathbf{R}$-vector of $(8, 8, 8)$ hopping greater than $5^{-4}$ eV when calculating $\langle S_z \rangle$.

We then analytically introduced the SOC operator to this basis. The total Hamiltonian is given by $\mathcal{H} = \mathcal{H}_0 + \mathcal{H}_{\text{SOC}}$, where the spin-orbit interaction is expressed as:

$$\mathcal{H}_{\text{SOC}} = \sum_{i \in \{\text{Cr, Sb}\}} \lambda_i \mathbf{L}_i \cdot \mathbf{S}_i = \sum_i \lambda_i \left[ L_i^z S_i^z + \frac{1}{2} \left( L_i^+ S_i^- + L_i^- S_i^+ \right) \right] \tag{1}$$

Here, $\lambda_i$ is the atomic spin-orbit coupling strength, $\mathbf{S}_i$ is the spin operator, and $\mathbf{L}_i$ is the orbital angular momentum operator projected onto the quantisation axis (which was set to $[0, 0, 1]$). For the antimony $p$-orbitals, assuming a local quantisation axis along $z$ and a basis of $(p_z, p_x, p_y)$,

the angular momentum matrices take the form:

$$L_x^p = \begin{pmatrix} 0 & 0 & i \\ 0 & 0 & 0 \\ -i & 0 & 0 \end{pmatrix}, \quad L_y^p = \begin{pmatrix} 0 & -i & 0 \\ i & 0 & 0 \\ 0 & 0 & 0 \end{pmatrix}, \quad L_z^p = \begin{pmatrix} 0 & 0 & 0 \\ 0 & 0 & -i \\ 0 & i & 0 \end{pmatrix} \tag{2}$$

For the chromium $d$-orbitals, in the basis $(d_{z^2}, d_{xz}, d_{yz}, d_{x^2-y^2}, d_{xy})$, the matrices are:

$$L_x^d = \begin{pmatrix} 0 & 0 & i\sqrt{3} & 0 & 0 \\ 0 & 0 & 0 & i & i \\ -i\sqrt{3} & 0 & 0 & 0 & 0 \\ 0 & -i & 0 & 0 & 0 \\ 0 & -i & 0 & 0 & 0 \end{pmatrix}, \quad L_y^d = \begin{pmatrix} 0 & -i\sqrt{3} & 0 & 0 & 0 \\ i\sqrt{3} & 0 & 0 & 0 & 0 \\ 0 & 0 & 0 & i & -i \\ 0 & 0 & -i & 0 & 0 \\ 0 & 0 & i & 0 & 0 \end{pmatrix}, \quad L_z^d = \begin{pmatrix} 0 & 0 & 0 & 0 & 0 \\ 0 & 0 & -i & 0 & 0 \\ 0 & i & 0 & 0 & 0 \\ 0 & 0 & 0 & 0 & -2i \\ 0 & 0 & 0 & 2i & 0 \end{pmatrix} \tag{3}$$

In the basis of the spin-projected Wannier states, the $L_\pm S_\mp$ terms of this interaction explicitly introduce off-diagonal mixing between the $|\uparrow\rangle$ and $|\downarrow\rangle$ states. This hybridises the Fermi sheets at the point of intersection. Consequently, SOC causes the spin-up and spin-down channels to mix, producing strongly spin-textured Fermi surface sheets (Fig. S4). Our implementation parametrises this spin-mixing interaction via the tunable parameters $\lambda_{\mathrm{Cr}}$ and $\lambda_{\mathrm{Sb}}$, allowing us to understand how the gap magnitude changes as a function of spin-orbit coupling. We note that the density of states near the Fermi level is dominated by contributions from the chromium $d$-electrons with a comparatively small amount of antimony $p$-weight. We therefore expect the effect of SOC to be relatively negligible, predominantly contributing to the hybridisation of up and down sheets, rather than causing a reconstruction of the Fermi surface, since chromium is a reasonably light transition metal.[14]

## 1.4 Magnetic Breakdown of the Fermi Surface

To determine exactly which cyclotron orbits the quasiparticles undertake upon introducing a magnetic field $B$, we must compare the energy scale associated with the SOC-induced band openings against the energy scale of the cyclotron orbits. While SOC lifts the nodal degeneracies and induces local Lifshitz transitions, the resulting hybridisation gaps ($E_g$) depend on the

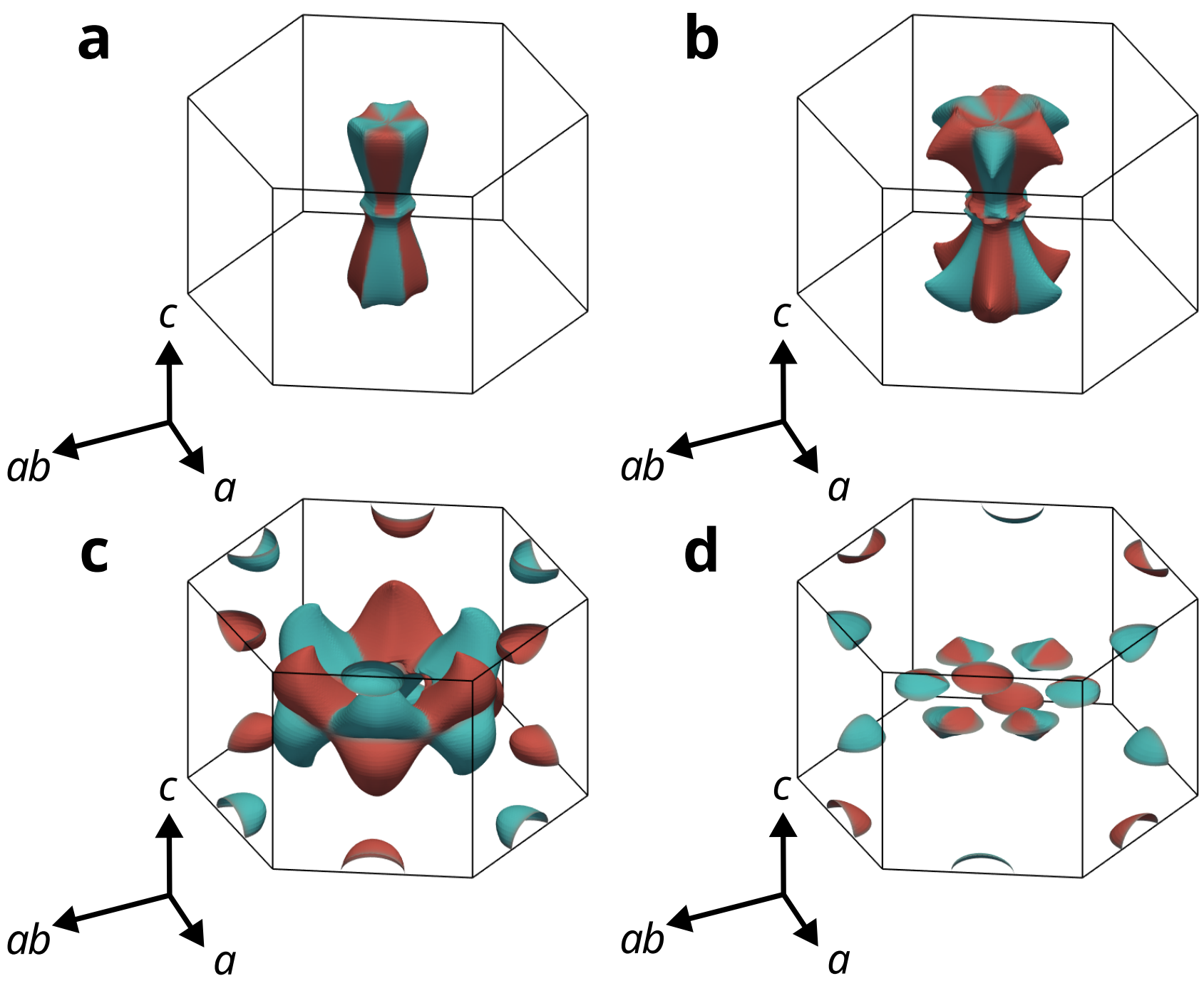


**Fig. S4. Spin-textured Fermi surface of CrSb including spin-orbit coupling.** The Fermi surface sheets of CrSb computed from a symmetrised Wannier-projected tight-binding model ($U = 3.5$ eV) with the analytical introduction of SOC ($\lambda_{\mathrm{Cr}} = 0.04$ eV, $\lambda_{\mathrm{Sb}} = 0.4$ eV). The colour scale represents the projection of $\langle S_z \rangle$ where red and blue indicate predominantly up-spin and down-spin states, respectively. **a,b** Centrally located sheets arising from hybridisation between the 'dogbone' sheets and **c,d** outer sheets from the hybridisation of up and down 'web' sheets. The sheets exhibit strong momentum-dependent spin texture, reflecting the hybridisation of the pristine altermagnetic bands at the nodal crossing points. The spin texture follows the $g$-wave symmetry of the altermagnetic symmetry of CrSb.

strength of the SOC interaction. In the case of CrSb, we expect SOC to be weak and therefore the gap size to be small.[14]

Under the large applied magnetic fields required for our measurements, it is feasible that quasiparticles may undergo magnetic breakdown, jumping across narrow momentum-space gaps between the hybridised Fermi surface sheets. To analyse our QO data, we therefore need to know the probability of a quasiparticle undergoing breakdown so that we can determine whether

the quasiparticles traverse the pristine or hybridised altermagnetic band structure.

The probability, $P$, of a quasiparticle successfully tunnelling across an avoided crossing is described by the standard Landau-Zener-type expression:[15,16]

$$P = \exp\left(-\frac{B_0}{B}\right) \tag{4}$$

where $B_0$ is the characteristic breakdown field.

To evaluate $B_0$, we extract the momentum-space orbital geometries from our tight-binding model along the principal axes of our measurements. As implemented in our numerical analysis, the breakdown field depends critically on the $k$-space distance between the unhybridised orbits, $\Delta k$, and the local curvature of the Fermi surface sheets at the avoided crossing. The semiclassical expression for the characteristic field is given by:[15,17]

$$B_0 = \frac{\pi\hbar}{2e}\sqrt{\frac{(\Delta k)^3}{\rho_1^{-1} + \rho_2^{-1}}}. \tag{5}$$

Here, $e$ is the elementary charge, $\hbar$ the reduced Planck constant, $\Delta k$ the distance in momentum-space between the two closest points on the respective quasiparticle orbits, and $\rho_1$ and $\rho_2$ are the local radii of curvature for orbit 1 and orbit 2 in $k$-space at those points.

Considering the pristine, unhybridised Fermi surface, the cross-sectional areas of the quasiparticle orbits induced by magnetic fields applied along the $c$-axis or $ab$ direction are strictly degenerate (see Fig. S5). This continuous degeneracy arises because fields oriented along these specific high-symmetry crystallographic directions induce cyclotron orbits that lie entirely within the symmetry-protected nodal planes, where the up-spin and down-spin Fermi surfaces coincide. Although the introduction of SOC lifts this degeneracy, for small SOC strengths, the resulting perturbed orbital paths remain virtually identical in both their overall topology and enclosed momentum-space area.

The most important geometry to consider, therefore, occurs when the applied magnetic field is tilted into the $c - a$ plane. In this configuration, the pristine altermagnetic Fermi surface

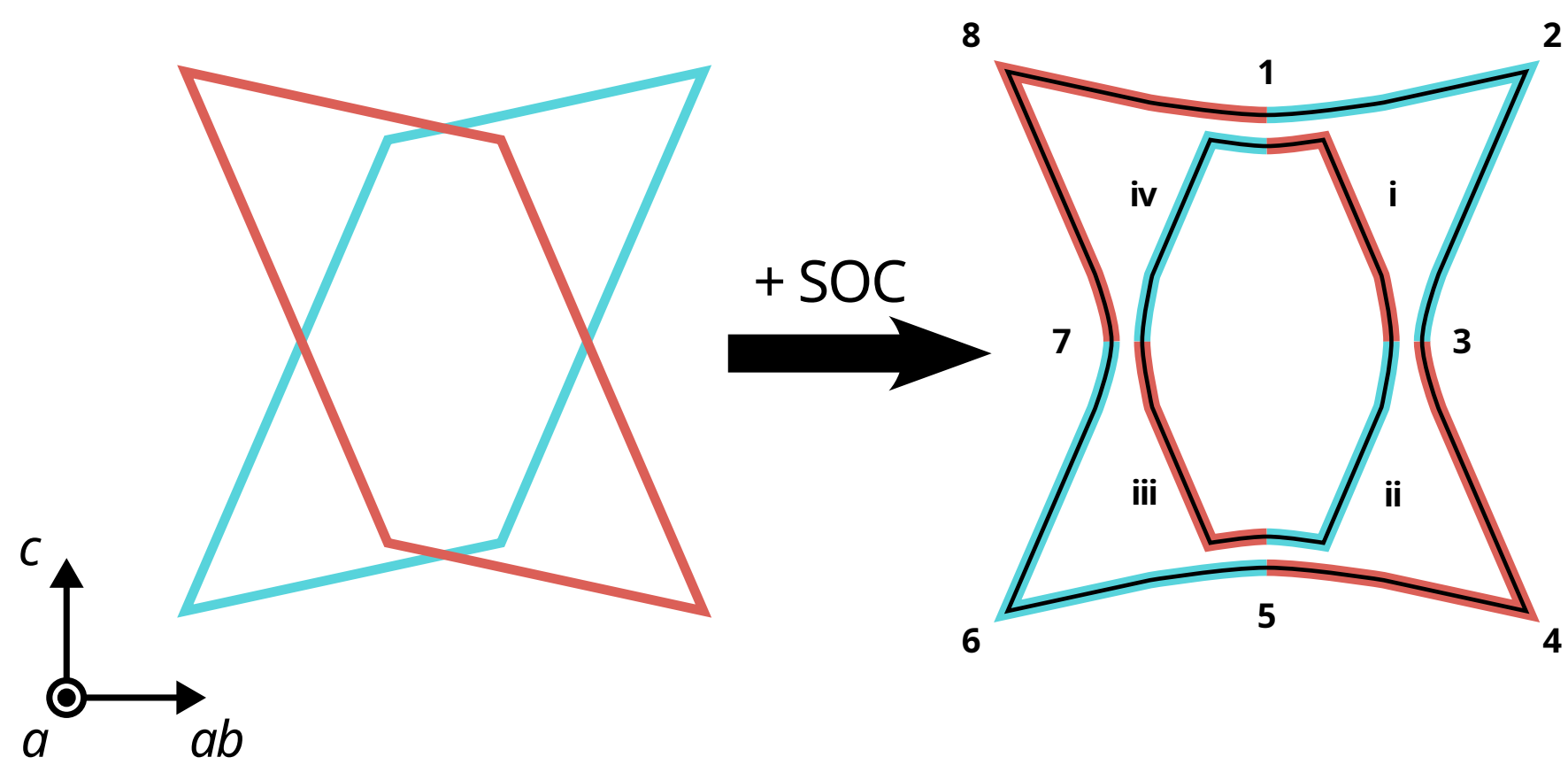


**Fig. S5. Schematic of magnetic breakdown at SOC-induced avoided crossings.** (Left) Pristine, unhybridised Fermi surface cross-sections as viewed down the $a$-axis. Along this direction, the cross-sections can be modelled as intersecting parallelograms, where spin-up (red) and spin-down (blue) sheets are mirror reflections of each other about the nodal planes. (Right) The introduction of SOC opens hybridisation gaps at intersection points. In high magnetic fields, quasiparticles follow the pristine path (e.g., 1-i-3-4-5-iii-7-8-1) via magnetic breakdown tunnelling (points 1, 3, 5, 7) rather than the hybridised outer path (1-2-3-4-5-6-7-8-1).

still yields degenerate QO frequencies. However, these identical frequencies now arise from physically distinct quasiparticle trajectories on the independent up-spin and down-spin sheets; these two separate orbits enclose the same area only because they are perfectly mapped onto one another by a mirror symmetry operating across the nodal plane.

The addition of SOC fundamentally alters this by forcing the crossing bands to hybridise at their points of intersection along the nodal plane. Because the bands physically merge and gap out, the two SOC-induced Fermi surfaces have a new geometry, forming two bands of mixed spin-texture. These sheets no longer enclose the same momentum-space area, and so if quasiparticles truly orbit these sheets, their respective QO frequencies would split into two distinct branches.

To compare these hybridised and pristine regimes, and to deduce the exact number of mag-

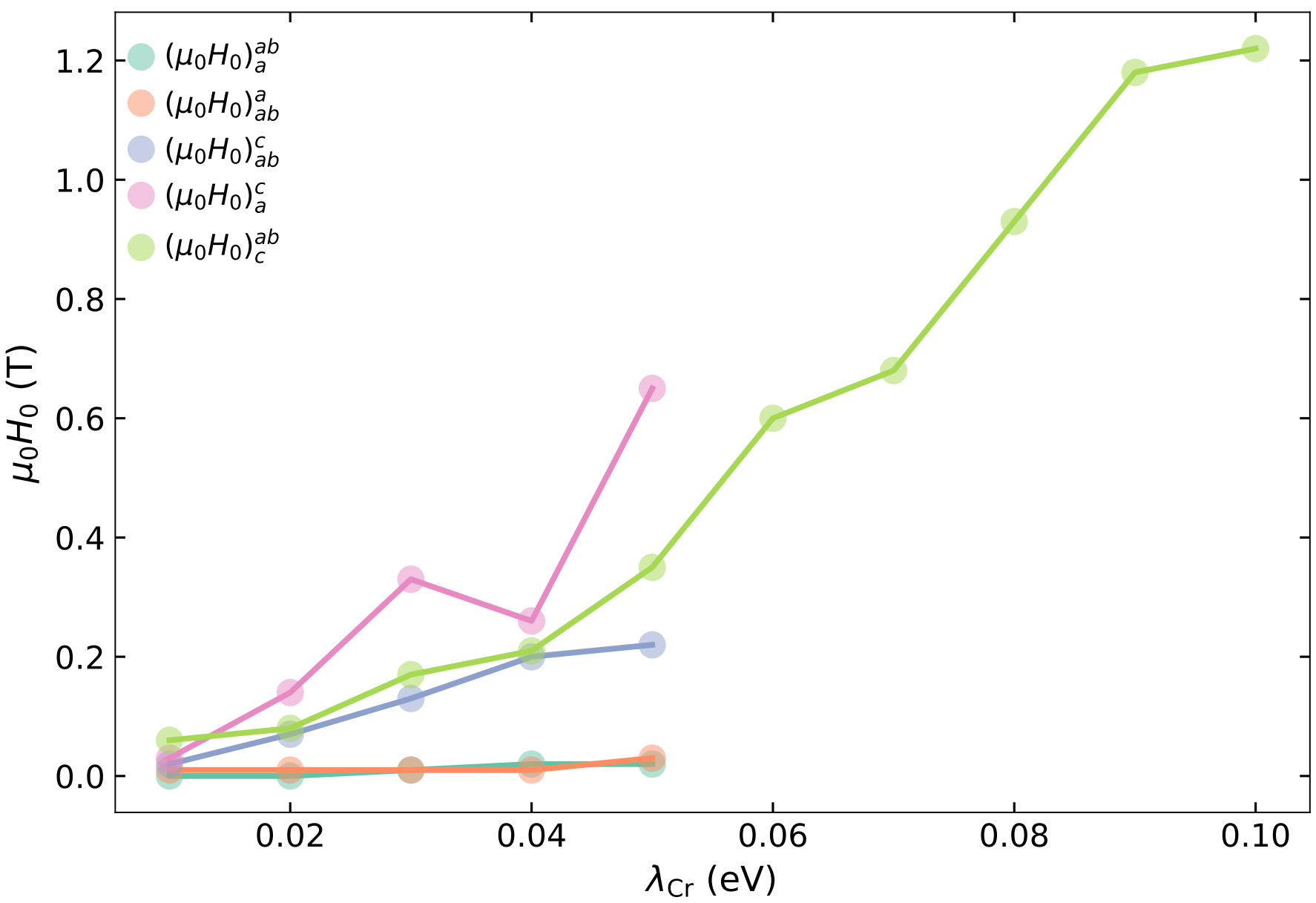


**Fig. S6. Dependence of the magnetic breakdown fields on spin-orbit coupling strength along different principal axes**. Calculated breakdown fields $(\mu_0 H_0)_i^j$ as a function of the Cr spin-orbit coupling parameter $\lambda_{Cr}$ (with $\lambda_{Sb}$ fixed at $10\lambda_{Cr}$). Subscripts denote the normal to the cyclotron orbit, while superscripts indicate the normal to the plane in which breakdown occurs. For orbits within the $a$- or $ab$-planes, data is extracted up to $\lambda_{Cr} = 0.05$ eV; beyond this coupling strength, hybridisation gaps at the dogbone ends prevent the completion of closed orbits, precluding the extraction of $(\mu_0 H_0)_{ab}^j$ or $(\mu_0 H_0)_a^j$. The breakdown fields remain small, even up to the largest values of spin-orbit coupling, revealing the high likelihood of quasiparticles undergoing breakdown orbits. Breakdown fields calculated using `py_FS`[5,6]

netic breakdown junctions a quasiparticle must successfully traverse to complete the equivalent of a pristine orbit, we can analyse a simplified 2D cross-section of the Fermi surface along the relevant nodal axis. In this projection, a minimal, symmetry-respecting model of the unhybridised Fermi surface sheets and their crossing points can be represented by two intersecting parallelograms (Fig. S5).

In Fig. S5, an orbit around the hybridised outer sheet corresponds to the path 1-2-3-4-5-6-7-8-1, whereas a path around the pristine, spin-split surface corresponds to the path 1-i-3-4-5-iii-7-8-1. To orbit the spin-split surface, a quasiparticle must tunnel to the neighbouring sheet at points 1, 3, 5, and 7. By symmetry, the probability of breakdown is equal at points 1 and 5. Similarly, the probability of breakdown at 3 and 7 is the same. We will denote the probability of breakdown at these two sets of points as $P_a^{ab}$ and $P_a^c$, respectively (where we use subscripts to denote the normal to the orbit, and superscripts indicate the normal to the plane in which breakdown occurs). The probability of an orbit around the pristine spin-split surface is $(P_a^{ab} P_a^c)^2$, whereas around the outer sheet it is $(P_a^{ab} - 1)^2 (P_a^c - 1)^2$.

We determined these breakdown probabilities between the two central dogbone pockets for varying strengths of SOC along these directions, as well as the other principal axes. Generally speaking, the SOC strength associated with chromium $d$-orbitals is of the order of 10s of meV,[18–20] whereas for antimony $p$-orbitals it is of the order of 100s of meV.[21,22] Therefore, we

| $\mu_0 H_{0c}^{ab}$ (T) | $\mu_0 H_{0a}^{ab}$ (T) | $\mu_0 H_{0a}^{c}$ (T) | $\mu_0 H_{0ab}^{a}$ (T) | $\mu_0 H_{0ab}^{c}$ (T) |
|---|---|---|---|---|
| 6.34 | 0.00 | 2.46 | 0.00 | 6.21 |

**Table S1: Calculated breakdown fields for $B$ oriented along high-symmetry crystallographic axes and planes for a DFT+$U$+SOC calculation performed at $U = 3.5$ eV.** Although the calculated breakdown fields for the DFT calculation are slightly larger than those determined from analytically adding SOC, they are sufficiently low such that at the high field strengths where we observe QOs, we overwhelmingly expect to observe primarily breakdown orbits.

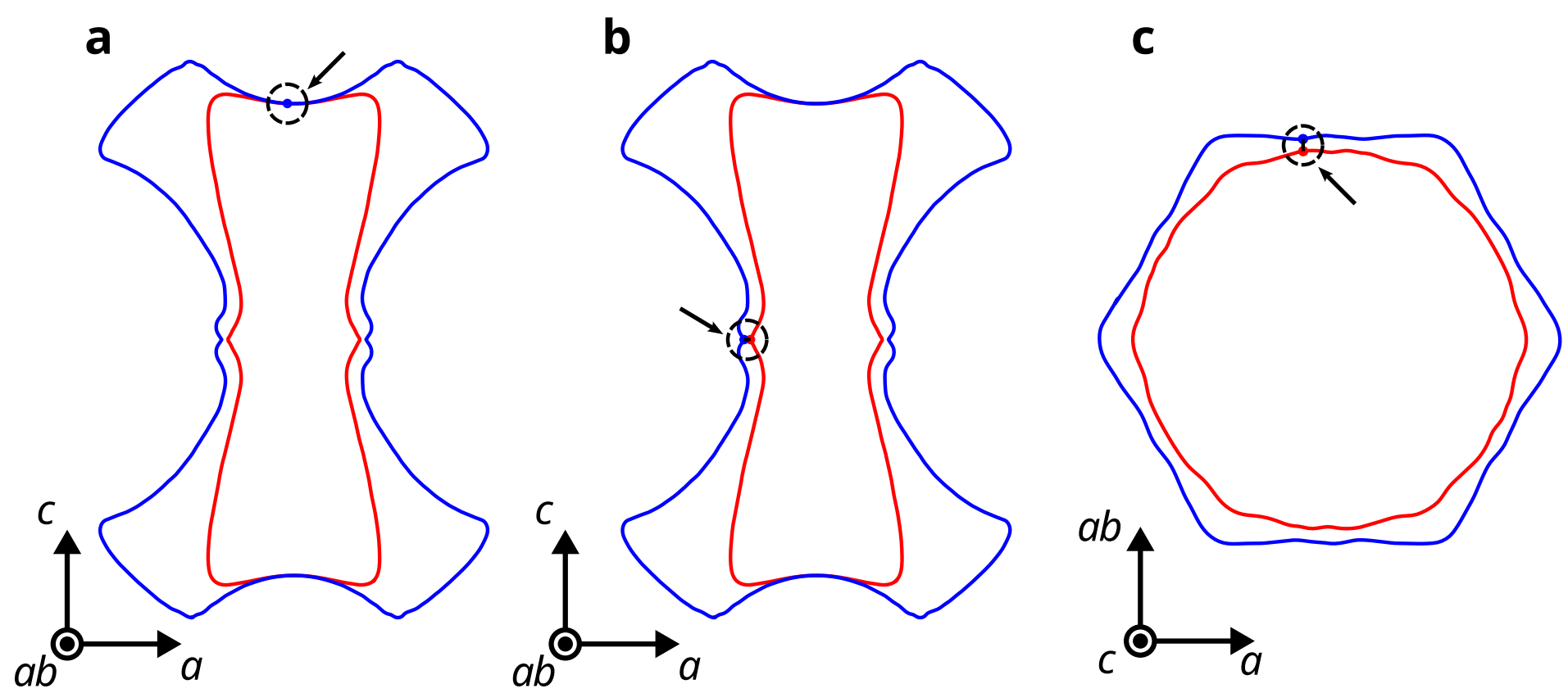


**Fig. S7. Visualisation of magnetic breakdown points in CrSb.** Cross-sections of the Fermi surface sheets in the (a,b) $ab - c$ and (c) $a - ab$ planes. These correspond to breakdown probabilities of $P^a_{ab}$, $P^c_{ab}$, and $\sim P^a_c$ (since the closest spacing in $k$-space lies slightly away from the plane defined by a normal in the $a$-direction).The red and blue lines represent the perimeter of two hybridised sheets calculated with spin-orbit coupling, which arise from the mixing of the 'dogbone' type surfaces in the up and down spin channels.

set $\lambda_{Sb} = 10\lambda_{Cr}$ in all these calculations.

We performed calculations with $0.01 \leq \lambda_{Cr} \leq 0.10$ eV, where above 0.05 eV we found that small openings appeared at the ends of the dogbone sheet meaning that quasiparticle orbits can no longer happen for fields along the $a$ or $ab$ directions and so we no do not extract $P^i_{ab}$ or $P^i_a$ for SOC couplings exceeding this.

Taking the breakdown fields for the $\lambda_{Cr} = 0.05$ eV, we get $\mu_0 H_0{}^{ab}_a = 0.02$ T and $\mu_0 H_0{}^c_a = 0.65$ T. Our QO measurements were mostly performed in the 30-41 T range. Therefore, assuming a field of 35 T we get $P^{ab}_a = 0.9994$ and $P^c_a = 0.9816$. Therefore, the ratio of breakdown orbits to orbits around the hybridised surface (given by $(P^{ab}_a P^c_a)^2 / \left[(P^{ab}_a - 1)^2 (P^c_a - 1)^2\right]$ is $8.7 \times 10^9$. This means that we expect the QO spectrum to be completely dominated by the breakdown orbits (similar to the case of ref.[23]), and so it should map well to the DFT-predicted spin-split surfaces.

We can perform the same analysis with a DFT+$U$+SOC calculation performed at $U$ =

3.5 eV (Table S1). Here we find that the breakdown fields are slightly larger for breakdown within the $a - ab$ plane when compared to our Wannier-derived model. However, for the orbit considered in Fig. S5, the sheets appear to coincide about the top of the Brillouin zone, giving $P_a^{ab} = 1$ and $P_a^c = 0.9321$, emphasising that the contribution of SOC at the Fermi level is incredibly small.

## 1.5 Simulated Orbits for Field Along $ab$ Direction

Directly mapping all experimentally observed spin-split frequency spectra to precise sections of the simulated Fermi surface is challenging. Subtle variations in Fermi surface topology, which density functional theory calculations may not fully capture, can result in substantially different expectations for the angular dependencies of the frequencies. In particular, while the frequency spectrum of the dogbone sheet is relatively straightforward, the complex geometry of the web sheet generates intricate frequency branches as a function of the applied field angle.

In the main text, we focus on the ≈3.5 kT frequencies observed for applied fields near the $ab$ direction, which we attribute to the dogbone Fermi pocket. This specific oscillatory component is well-separated from other oscillations in frequency space, allowing for a clean analysis of the contributing spin-split orbits.

Several factors enable us to confidently assign this frequency to an orbit on the dogbone sheet. Most notably, our measured spectrum reveals a continuous frequency branch that spans from ≈1.5 kT for fields along the $c$-axis to ≈3.5 kT along $ab$. In our simulations, this behaviour is only reproduced by the dogbone Fermi surface. Furthermore, across various values of $U$ or Fermi energy shifts, a ≈3.5 kT frequency contribution for fields applied along the $ab$ direction is exclusively associated with the dogbone sheet. The largest simulated frequency for the web sheet along $ab$ is ≈1.8 kT for $U = 3$ eV.

We analysed the frequency spectrum along the $ab$ direction for varying Fermi energy shifts

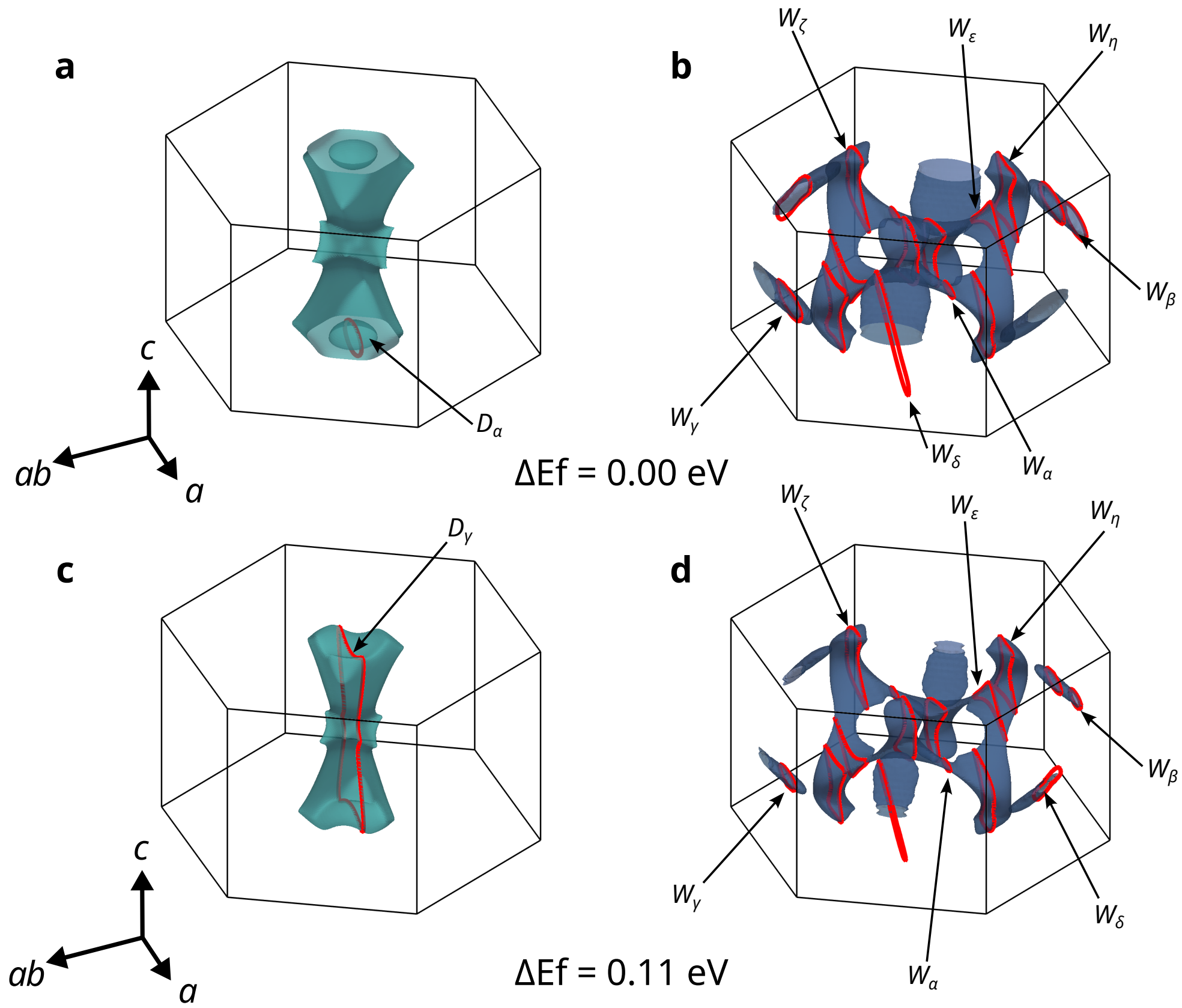


**Fig. S8. Predicted orbits for field along the $ab$ direction for different Fermi surface shifts.** The dogbone Fermi surface sheet and the web Fermi surface sheet at $\Delta E_\mathrm{f}$ = 0.00 eV (a, b) and $\Delta E_\mathrm{f}$ = 0.11 eV (c, d). Labelled red lines indicate the extremal orbits for this field direction. Fermi surface shifts are given for the dogbone sheet, the web sheet is shifted accordingly to keep the overall carrier concentration constant. When $\Delta E_\mathrm{f}$ = 0.11 eV the dogbone sheet undergoes a Lifshitz transition, becoming a closed 3D pocket. The QO frequencies were calculated using SKEAF,[24] see Tables in text below.

up to 0.16 eV (larger than that used in the main text, where a shift of 0.11 eV was imposed). At $\Delta E_f$ = 0.11 eV, there is a Lifshitz transition of the dogbone sheet which closes to form a large, closed orbit with a frequency ≈3 kT. The orbits associated with the web sheet produce oscillatory frequencies of at most 0.83 kT in this model. The quasiparticle orbits are defined in Figures S8 & S9.

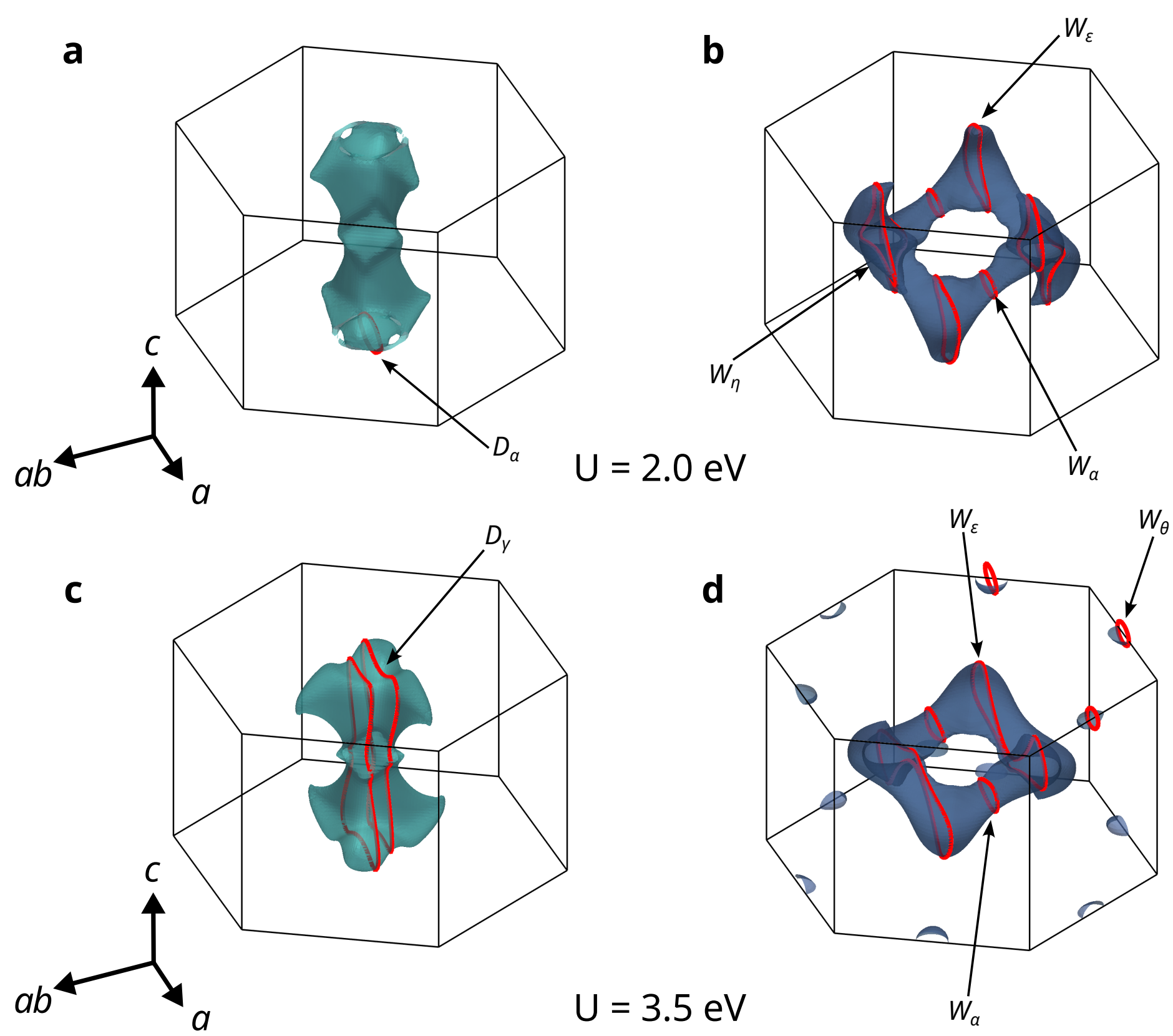


**Fig. S9. Predicted orbits for field along the $ab$ direction for different Hubbard $U$ values.** The dogbone and web Fermi surface sheets determined for $U = 2.0$ eV (a, b) and $U = 3.5$ eV (c, d). Correlations induce a Lifshitz transition for both the web and dogbone sheets, causing the initially open dogbone to sheet to close about the edges of the Brillouin zone, while small spherical pockets form in the web sheet. QO frequencies from these orbits were calculated using SKEAF,[24] see Tables in text below.

We performed the same analysis for our calculations, including $d$-orbital correlations through a Hubbard $U$ parameter. Sufficient correlations induce a Lifshitz transition in both the dogbone and web sheets. The dogbone sheet closes for $U > 3$ eV, whereas small spherical pockets are present around the edge of the Brillouin zone for the web sheet.

**Shift = 0.00 eV**

| Dogbone | | Web | |
|---|---|---|---|
| Orbit | Frequency (kT) | Orbit | Frequency (kT) |
| $D_\alpha$ | 0.368 kT | $W_\alpha$ | 0.062 kT |
| | | $W_\beta$ | 0.376 kT |
| | | $W_\gamma$ | 0.412 kT |
| | | $W_\delta$ | 0.477 kT |
| | | $W_\epsilon$ | 0.634 kT |
| | | $W_\zeta$ | 0.777 kT |
| | | $W_\eta$ | 0.826 kT |

**Shift = 0.05 eV**

| Dogbone | | Web | |
|---|---|---|---|
| Orbit | Frequency (kT) | Orbit | Frequency (kT) |
| $D_\alpha$ | 0.484 kT | $W_\alpha$ | 0.048 kT |
| | | $W_\beta$ | 0.251 kT |
| | | $W_\gamma$ | 0.297 kT |
| | | $W_\delta$ | 0.417 kT |
| | | $W_\epsilon$ | 0.486 kT |
| | | $W_\zeta$ | 0.729 kT |
| | | $W_\eta$ | 0.744 kT |

**Shift = 0.11 eV**

| Dogbone | | Web | |
|---|---|---|---|
| Orbit | Frequency (kT) | Orbit | Frequency (kT) |
| $D_\beta$ | 3.047 kT | $W_\alpha$ | 0.034 kT |
| | | $W_\beta$ | 0.105 kT |
| | | $W_\gamma$ | 0.170 kT |
| | | $W_\delta$ | 0.288 kT |
| | | $W_\epsilon$ | 0.351 kT |
| | | $W_\zeta$ | 0.676 kT |
| | | $W_\eta$ | 0.660 kT |

**Shift = 0.16 eV**

| Dogbone | | Web | |
|---|---|---|---|
| Orbit | Frequency (kT) | Orbit | Frequency (kT) |
| $D_\beta$ | 2.589 kT | $W_\gamma$ | 0.063 kT |
| | | $W_\delta$ | 0.670 kT |
| | | $W_\epsilon$ | 0.302 kT |
| | | $W_\zeta$ | 0.634 kT |
| | | $W_\eta$ | 0.598 kT |

**$U$ = 2.0 eV**

| Dogbone | | Web | |
|---|---|---|---|
| Orbit | Frequency (kT) | Orbit | Frequency (kT) |
| $D_\alpha$ | 0.417 kT | $W_\alpha$ | 0.167 kT |
| | | $W_\zeta$ | 0.905 kT |
| | | $W_\eta$ | 0.980 kT |

**$U$ = 3.0 eV**

| Dogbone | | Web | |
|---|---|---|---|
| Orbit | Frequency (kT) | Orbit | Frequency (kT) |
| $D_\beta$ | 3.489 kT | $W_\alpha$ | 0.238 kT |
| | | $W_\zeta$ | 0.881 kT |
| | | $W_\eta$ | 1.874 kT |

**$U$ = 3.5 eV**

| Dogbone | | Web | |
|---|---|---|---|
| Orbit | Frequency (kT) | Orbit | Frequency (kT) |
| $D_\gamma$ | 3.578 kT | $W_\alpha$ | 0.153 kT |
| | | $W_\epsilon$ | 1.770 kT |
| | | $W_\theta$ | 0.272 kT |

$U$ **= 4 eV**

| Dogbone | | Web | |
|---|---|---|---|
| Orbit | Frequency (kT) | Orbit | Frequency (kT) |
| $D_\gamma$ | 3.459 kT | $W_\alpha$ | 0.159 kT |
| | | $W_\epsilon$ | 1.241 kT |
| | | $W_\zeta$ | 0.596 kT |
| | | $W_\theta$ | 0.844 kT |

We note that our models incorporating SOC predict that the SOC-hybridised web sheet may host a large quasiparticle orbit with an associated oscillatory frequency of ≈3.4 kT (Fig. S10). However, as discussed previously, the small magnetic breakdown fields associated with the Fermi surface of CrSb mean that the probability of observing frequencies derived from such an orbit (at the applied fields where quantum oscillations are resolved) is vanishingly small compared to oscillations generated by quasiparticles traversing the spin-split Fermi surface. Indeed, the expected angular dependence of this specific orbit (very flat with angle, see Figs S12 and S14) does not resemble any frequency branch resolved in our measurements. We therefore conclude that such a large web orbit is not observed experimentally.

Our argumentation in the main text – that we have resolved the $g$-wave spin texture of altermagnetic CrSb through rotational quantum oscillation quasiparticle spectroscopy – is largely premised on the geometric properties of the ≈3.5 kT frequency spectra. We argue in the main text, that in the nodal $c - a$ rotation plane our observation of this frequency branch having a four-fold rotational profile (i.e. identical repetition of frequency versus angle every 90°) is as expected for symmetry-enforced spin degeneracy. By contrast, rotating the magnetic field through the antinodal $c - ab$ plane, two frequency traces are now observed around the 3-4 kT range, each of which only repeats every 180°. This reduction of symmetry is exactly as expected in the case of each of these frequency versus angle traces corresponding to opposite quasiparticle spin species (one up, the other down).

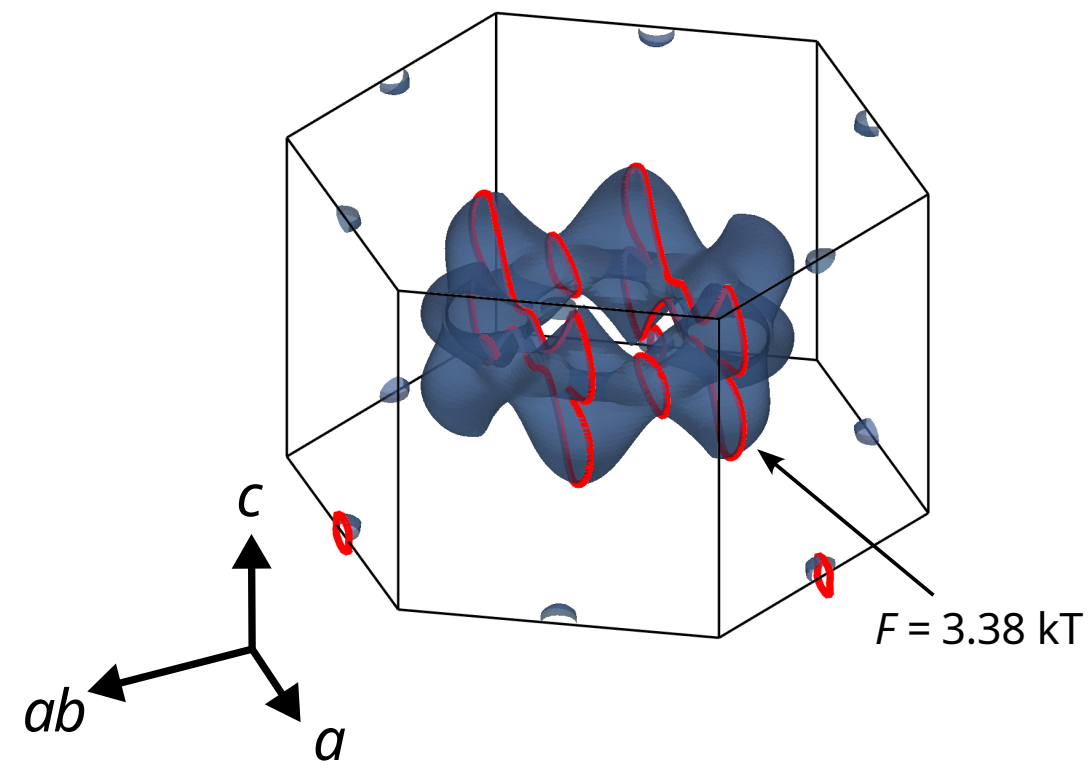


**Fig. S10. Hypothetical quantum oscillation orbit with a frequency of 3.4 kT from the SOC hybridised Fermi surface sheets of CrSb with $U = 3.5$ eV.** The SOC hybridised web sheet of CrSb hosts an orbit with an associated frequency of 3.4 kT for fields near the $ab$ direction. However, this orbit vanishes when fields are directly applied along $ab$, and we expect that the large fields required to observe quantum oscillations will induce breakdown orbits around the unhybridised sheets. It is also expected to possess a frequency versus angle profile (Fig. S12) very different to that observed experimentally (Main Fig. 2). We conclude that this hypothetical orbit path is not observed experimentally.

In this section, we have shown in detail that the electron (web) Fermi pocket of CrSb is strongly expected – for a broad range of calculational parameters, and especially those that closely match the experimental QO data – to yield a large number of frequencies for fields oriented close to $ab$. However, importantly, none of these frequency components are ever expected to be greater than 2 kT. This lends strong confidence to our attribution of the >3 kT orbits to the hole (dogbone) Fermi pocket.

We should note that, while this argument rules out frequency components in the range 3-4 kT emanating from fundamental orbits about the web sheet, in principle it leaves open the possibility that higher harmonics of web sheet orbits might provide an alternative explanation for their origin. However, an inspection of their effective cyclotron masses strongly rules out this scenario. In Extended Data Fig. 9 a Lifshitz-Kosevich temperature study is presented. Nu-

merous frequency branches are resolved, ranging from under 1 kT to almost 4 kT. Importantly, they all yield very similar effective masses ($\sim$ twice that of the bare electron mass). It is a well known property of the de Haas van Alphen effect that a second harmonic frequency component will possess twice the effective mass of its corresponding fundamental.[25] This extends similarly to higher harmonics – the $n^{\text{th}}$ harmonic possesses $n$ times the mass of the fundamental. However, the fact that all of the frequency components in Extended Data Fig. 9 exhibit very similar temperature-damping profiles strongly argues that each FFT peak corresponds to distinct fundamental orbits. For the numerous reasons outlined in this section, we conclude that the 3-4 kT frequency traces we focus on in the main text are almost certainly from the dogbone Fermi pocket identified in DFT calculations.

# 2 Summary

In summary, by systematically including the effects of correlations and SOC, we have reconciled QO measurements of the metallic altermagnet CrSb with DFT predictions. We have determined that by introducing a Hubbard correlation of $U > 2.5$ eV, we can induce a Lifshitz transition of the Fermi surface into a closed 'dogbone' shape, physically motivating the shift of the band edges applied in the main text. Although the addition of SOC lifts the altermagnetic nodal degeneracies, the induced gaps between Fermi surface sheets are very small as determined by both DFT+$U$+SOC calculations alongside analytically modelling SOC via a tight-binding Hamiltonian extracted from our DFT+$U$ calculations. Using a semiclassical Landau-Zener framework, we have calculated the characteristic magnetic breakdown fields associated with these gaps. The calculated values of the breakdown fields are typically orders of magnitude smaller than the experimentally applied fields in our QO study, meaning the tunnelling probability approaches unity. This confirms that quasiparticles readily undergo magnetic breakdown across the SOC-induced gaps, demonstrating that the observed QO frequencies di-

rectly map to the pristine, unhybridised, $g$-wave spin-split Fermi surface. We have demonstrated that the ≈3.5 kT frequency resolved in our measurements for fields along the $ab$ direction can be attributed to the dogbone Fermi pocket, allowing us to focus our analysis on the spin-split frequencies associated with orbits around this sheet, which can easily be differentiated from other orbits because they are isolated in frequency space. This confirms that we can determine the bulk symmetry of the altermagnetic order parameter by resolving the location of the nodal planes where the Fermi surface sheets intersect, and places CrSb (alongside other altermagnetic metals more generally) within the pantheon of materials expected to exhibit non-Onsager oscillatory behaviour at high fields.[26–30]

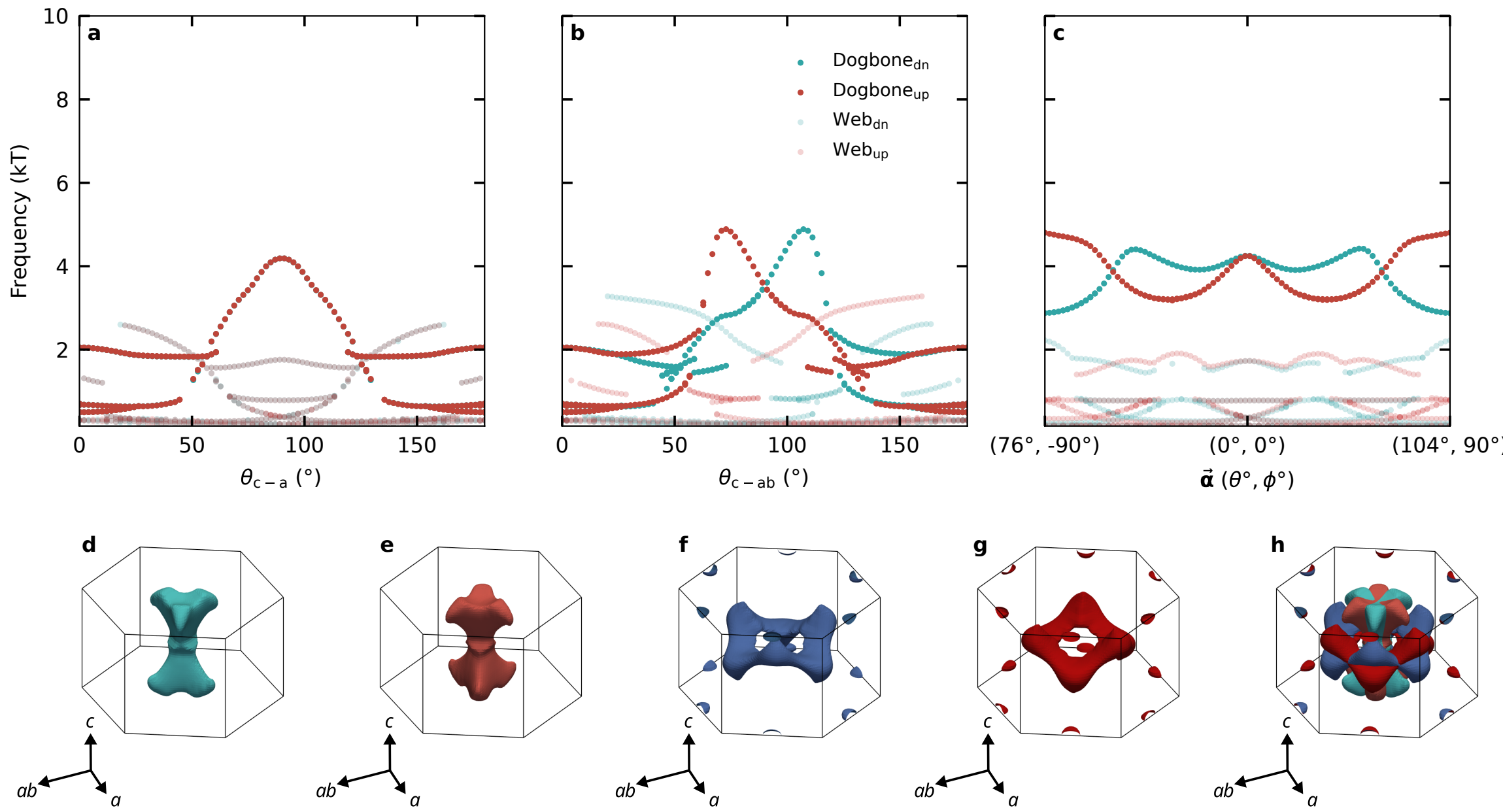


**Fig. S11. Angular dependence of quantum oscillation frequencies for the DFT+$U$ calculated altermagnetic Fermi surfaces.** Here we exclude SOC and set $U = 3.5$ eV. (a-c) Calculated dHvA frequencies as a function of field orientation for fields tilted in the $c-a$, $c-ab$, and the low symmetry $\vec{\alpha}(\theta, \phi)$ plane. The red and blue markers represent frequencies from the up-spin and down-spin channels, respectively, for the central 'dogbone' (solid) and outer 'web' (faded) sheets. (d-g) Corresponding spin-polarised Fermi surface sheets and (h) the complete Fermi surface. The perfect mapping of blue and red branches via mirror symmetry across the nodal planes results in the degeneracy observed when the field is applied within $c-a$ plane or along the $ab$ or $c$ axes. QO frequencies calculated using `SKEAF`.[24]

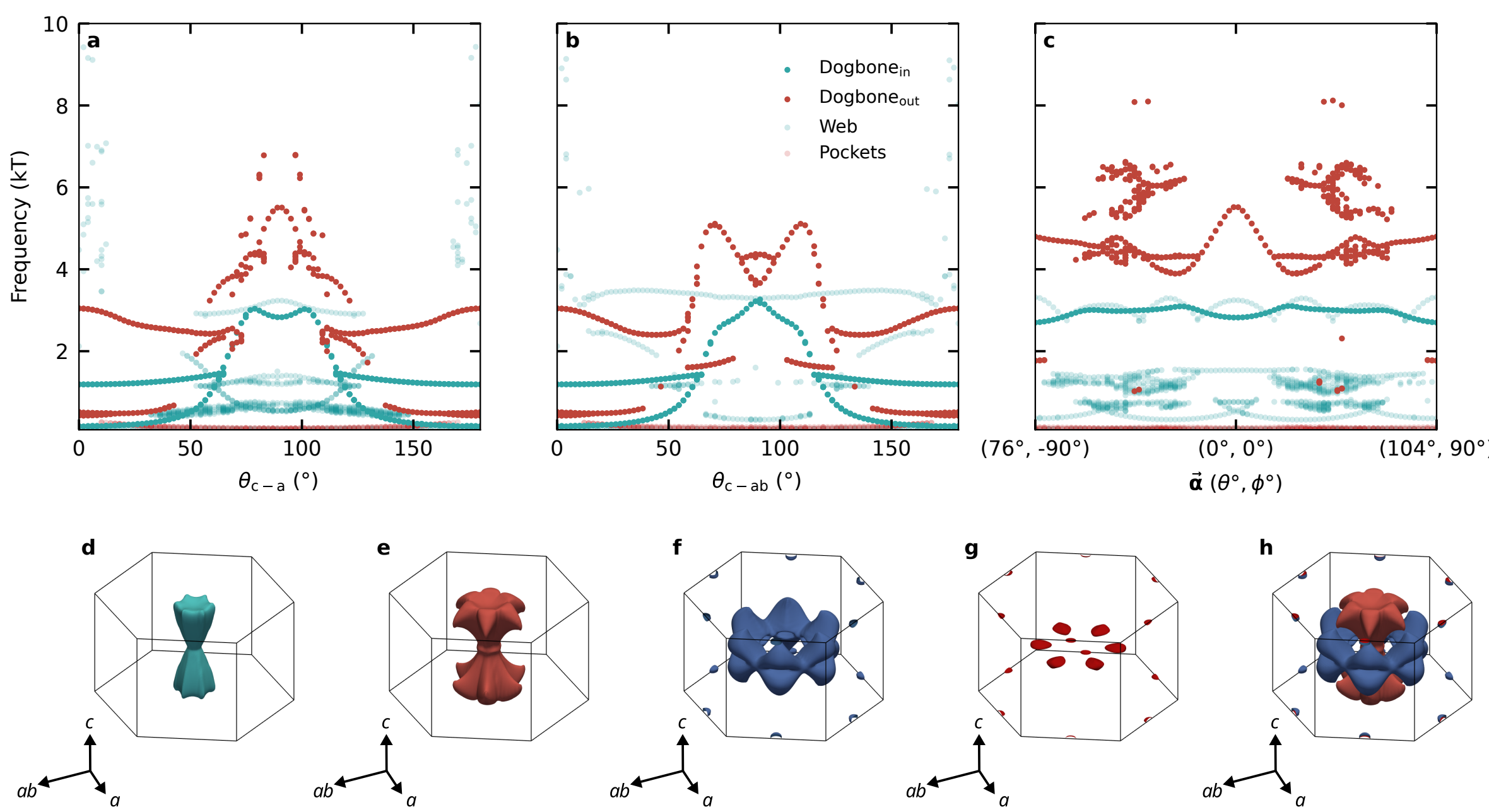


**Fig. S12. Angular dependence of quantum oscillation frequencies for the DFT+$U$+SOC calculated altermagnetic Fermi surfaces with $U$ = 3.5 eV.** (a-c) QO frequencies as a function of field orientation for fields tilted in the $c - a$, $c - ab$, and the low symmetry $\vec{\alpha}(\theta, \phi)$ plane. The blue marker represents frequencies from the central 'dogbone', whereas red corresponds to the outer 'dogbone'. Faded markers correspond to oscillation frequencies that arise from the hybridised 'web' sheets. The (d) inner 'dogbone', (e) outer 'dogbone', (f) large 'web', (g) small pockets arising from the SOC-induced hybridisation of the 'web' and (h). QOs arising from the hybridised sheets are no longer degenerate.

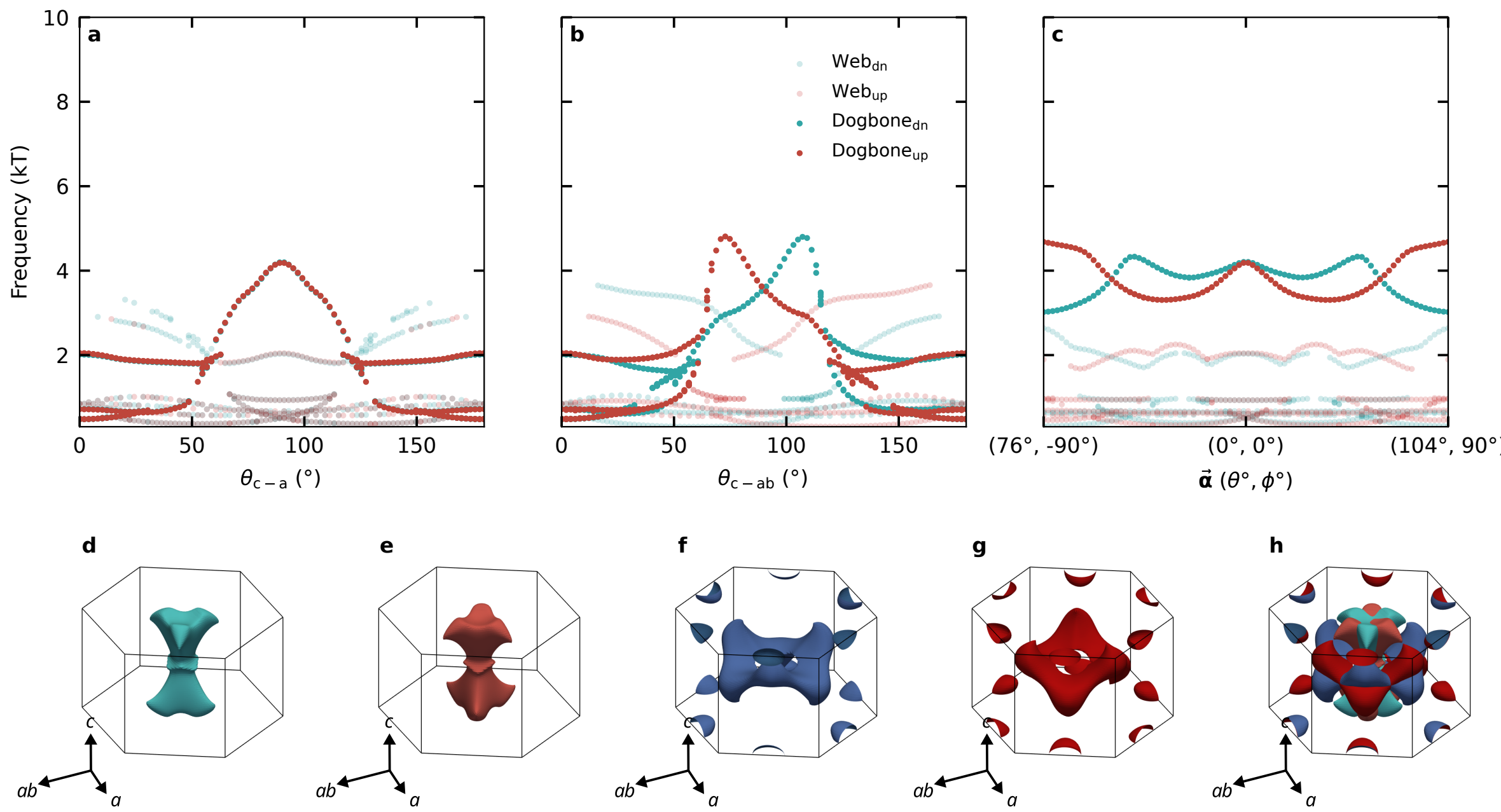


**Fig. S13. Angular dependence of quantum oscillation frequencies for the Wannier-decomposed altermagnetic Fermi surface.** Calculated frequencies are derived from a symmetrised Wannier tight-binding model ($U = 3.5$ eV) in the absence of spin-orbit coupling. (a-c) Calculated dHvA frequencies as a function of field orientation for fields tilted in the $c-a$, $c-ab$, and the low-symmetry $\vec{\alpha}(\theta, \phi)$ planes. The red and blue markers represent frequencies from the up-spin and down-spin channels, respectively, for the central 'dogbone' (solid) and outer 'web' (faded) sheets. (d-g) Corresponding spin-polarised Fermi surface sheets and (h) the complete unhybridised Fermi surface. The perfect mapping of blue and red branches via mirror symmetry across the nodal planes results in the degeneracy observed when the field is applied within the $c - a$ plane or along the $ab$ or $c$ axes.

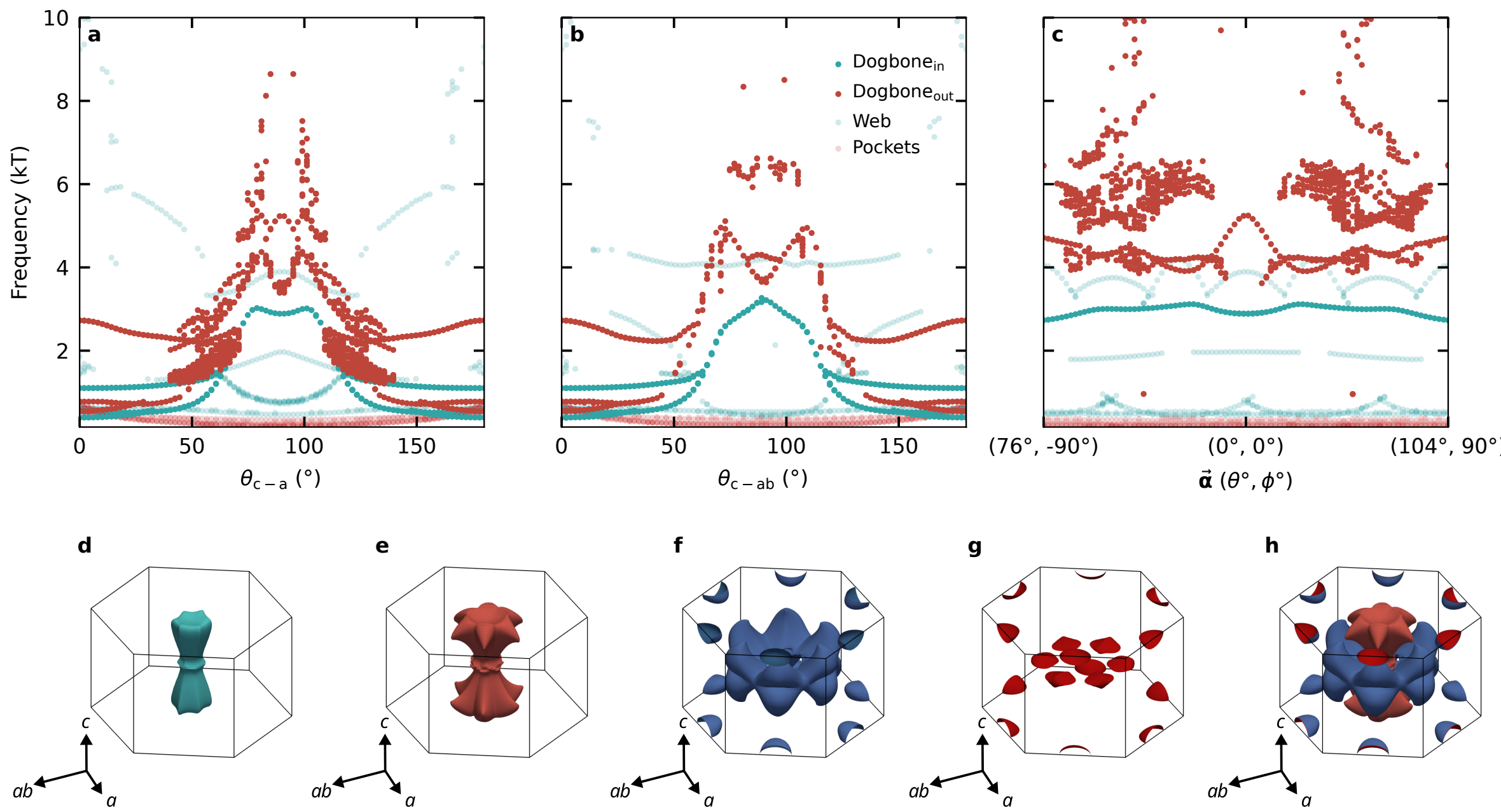


**Fig. S14. Angular dependence of quantum oscillation frequencies for the hybridised Wannier-decomposed Fermi surface with SOC based on a DFT+$U$ calculation with $U = 3.5$ eV.** Here we have analytically added SOC to our Wannier tight-binding mode ($\lambda_{\mathrm{Cr}} = 0.04$ eV, $\lambda_{\mathrm{Sb}} = 0.4$ eV). (a-c) QO frequencies as a function of field orientation for fields tilted in the $c - a$, $c - ab$, and the low-symmetry $\vec{\alpha}(\theta, \phi)$ planes. Blue markers represent frequencies from the inner 'dogbone' sheet, while red markers correspond to the outer 'dogbone'. Faded markers indicate frequencies arising from the hybridised 'web' sheets. (d-h) Corresponding SOC-induced Fermi surface sheets: (d) the inner 'dogbone', (e) the outer 'dogbone', (f) the large reconstructed 'web', and (g,h) the small pockets arising from the hybridisation of the 'web' sheets. Due to SOC-induced band mixing, frequencies arising from these hybridised sheets are no longer degenerate.

# References


1. Blaha, P. *et al.* WIEN2k: An APW+lo program for calculating the properties of solids. *J. Chem. Phys.* **152**, 074101 (2020).

2. Snow, A. I. Neutron Diffraction Investigation of the Atomic Magnetic Moment Orientation in the Antiferromagnetic Compound CrSb. *Phys. Rev.* **85**, 365–365 (1952).

3. Anisimov, V. I., Solovyev, I. V., Korotin, M. A., Czyżyk, M. T. & Sawatzky, G. A. Density-functional theory and NiO photoemission spectra. *Physical Review B* **48**, 16929–16934 (1993).

4. Czyżyk, M. T. & Sawatzky, G. A. Local-density functional and on-site correlations: The electronic structure of La 2 CuO 4 and LaCuO 3. *Physical Review B* **49**, 14211–14228 (1994).

5. Weinberger, T. Py_FS, GitHub repository [https://github.com/TheoWeinberger/py_FS] (2023). URL `https://github.com/TheoWeinberger/py_FS`.

6. Eaton, A. G. *et al.* Quasi-2D Fermi surface in the anomalous superconductor $UTe_2$. *Nat. Commun.* **15**, 223 (2024).

7. Wilde, M. A. *et al.* Symmetry-enforced topological nodal planes at the Fermi surface of a chiral magnet. *Nature* **594**, 374–379 (2021).

8. Huber, N. *et al.* Network of Topological Nodal Planes, Multifold Degeneracies, and Weyl Points in CoSi. *Phys. Rev. Lett.* **129**, 026401 (2022).

9. Terashima, T. *et al.* Altermagnetic spin-split Fermi surfaces in CrSb revealed by quantum oscillation measurements (2026). arXiv:`2601.19105`.

10. Mostofi, A. A. *et al.* An updated version of wannier90: A tool for obtaining maximally-localised Wannier functions. *Computer Physics Communications* **185**, 2309–2310 (2014).

11. Coh, S., Vanderbilt, D. & Cole, T. Python Tight Binding (PythTB). Zenodo (2025).

12. Tsirkin, S. S. High performance Wannier interpolation of Berry curvature and related quantities with WannierBerri code. *npj Computational Materials* **7**, 33 (2021).

13. Zhi, G.-X., Xu, C., Wu, S.-Q., Ning, F. & Cao, C. WannSymm: A symmetry analysis code for Wannier orbitals. *Computer Physics Communications* **271**, 108196 (2022).

14. Yang, G. *et al.* Three-dimensional mapping of the altermagnetic spin splitting in CrSb. *Nat. Commun.* **16**, 1442 (2025).

15. Chambers, R. G. Magnetic breakdown in real metals. *Proceedings of the Physical Society* **88**, 701–715 (1966).

16. Carter, J.-M., Podolsky, D. & Kee, H.-Y. Mean-field analysis of quantum oscillations, Fermi-surface topology, and magnetic breakdown in high- T c cuprates. *Phys. Rev. B* **81**, 064519 (2010).

17. Daschner, M. & Grosche, F. M. Electron-Hole Tunnelling Probed in de Haas - van Alphen Oscillations in the (Double) Dirac Semimetal $NbTe_4$ (2025). arXiv:2507.02579.

18. Yadav, R. *et al.* Electronic excitations and spin interactions in chromium trihalides from embedded many-body wavefunctions. *npj 2D Materials and Applications* **8**, 56 (2024).

19. Wang, Y. *et al.* Ballistic transport of orbital current from chromium. *Phys. Rev. B* **112**, 184408 (2025).

20. Lee, I. *et al.* Spin-orbit coupling controlled two-dimensional magnetism in chromium trihalides. *Physical Review B* **113**, 014401 (2026).

21. Rudenko, A. N., Katsnelson, M. I. & Roldán, R. Electronic properties of single-layer antimony: Tight-binding model, spin-orbit coupling, and the strength of effective Coulomb interactions. *Physical Review B* **95**, 081407 (2017).

22. Chadi, D. J. Spin-orbit splitting in crystalline and compositionally disordered semiconductors. *Physical Review B* **16**, 790–796 (1977).

23. Guo, C. *et al.* Quasi-symmetry-protected topology in a semi-metal. *Nat. Phys.* **18**, 813–818 (2022).

24. Rourke, P. & Julian, S. Numerical extraction of de Haas–van Alphen frequencies from calculated band energies. *Comput. Phys. Commun.* **183**, 324–332 (2012).

25. Shoenberg, D. *Magnetic Oscillations in Metals* (Cambridge University Press, Cambridge, UK, 1984). URL `https://doi.org/10.1017/CBO9780511897870`.

26. Weinberger, T. I. *et al.* Quantum Interference between Quasi-2D Fermi Surface Sheets in $UTe_2$. *Phys. Rev. Lett.* **132**, 266503 (2024).

27. Kartsovnik, M. V. *et al.* Direct Observation of the Magnetic-Breakdown Induced Quantum Interference in the Quasi-Two-Dimensional Organic Metal $\kappa$-(BEDT- TTF)$_2$CU(NCS)$_2$. *Phys. Rev. Lett.* **77**, 2530–2533 (1996).

28. Singleton, J. Studies of quasi-two-dimensional organic conductors based on BEDT-TTF using high magnetic fields. *Rep. Prog. Phys.* **63**, 1111 (2000).

29. Weinberger, T. I. *et al.* Pressure-enhanced $f$-electron orbital weighting in $UTe_2$ mapped by quantum interferometry. *Commun. Phys.* **8** (2025).

30. Huber, N. *et al.* Quantum oscillations of the quasiparticle lifetime in a metal. *Nature* **621**, 276–281 (2023).
