# Supplementary material for: 3D bulk-resolved g-wave altermagnetic order parameter in CrSb
Source: Nature. 2026 Aug 26;656(8129):854–60. doi: 10.1038/s41586-026-10902-z (PMC13518244; doi:10.1038/s41586-026-10902-z)
Supplement: Supplementary file 2 [file 41586_2026_10902_MOESM2_ESM.pdf]

nature portfolio

Peer Review File

3D bulk-resolved g-wave altermagnetic order parameter in CrSb

Corresponding Author: Dr Alexander Eaton

Any redactions in this file are there to maintain patient confidentiality, the confidentiality of unpublished data, or to remove third-party material.

This file contains all reviewer reports in order by version, followed by all author rebuttals in order by version.

Version 0:

Reviewer comments:

Referee #1

(Remarks to the Author)
Recently, there have been many reports on the electronic band, magnetic structure, electronic transport properties of CrSb, which is the typical example of altermagnets, as a novel, third class of magnet, alongside with ferro- and antiferromagnets. Especially, the occurring of a spin-splitting electronic bands caused by the atomic exchange interaction in this material has attracted much attention. In this manuscript, Mengmeng Long et al. confirmed that this altermagnetic band structure leads to the loss of mirror symmetry for each spin-split Fermi sheet away from highly symmetric nodal orientations; the difference between up and down spins follows the profile of the spherical harmonic, analogous to a g-orbital of the H atom, by using the magnetic quantum oscillation (QO) measurements with different orientations of magnetic field relative to the low and high-symmetry directions of its Brillouin zone. These results are interesting for understanding details of the spin-splitting in the metallic altermagnets, and the experimental data are reliable. However, whether the reported results, or the used method (the QO measurements by means of cantilever at high magnetic field), or the studied material are not so new. I think that the manuscript is inadequate to be published in Nature, while I agree to recommend it to be published in Nature Phys. or Nature Commun.

Referee #2

(Remarks to the Author)
Mengmeng Long and colleagues report the growth and characterization of high-quality single-crystalline CrSb and the investigation of its Fermi surface properties by quantum oscillation experiments with the goal of demonstrating the predicted altermagnetic g-wave symmetry of the spin splitting experimentally.
The topic is of great current interest, and a conclusive demonstration of the altermagnetic spin splitting in a good metal would be an important step forward. However, results like these being awaited by the community may have created some pressure towards early submission.

The paper is written in clear language, and the work's significance is well presented. The experimental data seem well-suited for the task at hand.
However, the manuscript has severe shortcomings in its current form, so there is no unambiguous demonstration of the altermagnetic g-wave spin splitting.
The main problems lie at the heart of the quantum oscillatory study: to demonstrate the unconventional g-wave spin structure, a complete Fermi surface determination is mandatory, because otherwise it remains unclear if the specific orbits the authors singled out for their case are really the ones they assume they are. However, this has not been done at all. The experimental data may well be sufficient for the task; however, we cannot tell because no attempt is made to assign all orbits to the Fermi surface.
The presented DFT calculation for the Fermi surface is insufficient:
(i) Most severely, the authors completely neglect spin-orbit coupling (SOC) in their calculations and discussions. I do not see any justification for that: SOC is expected to alter the band splittings on an energy scale in the same ballpark as the altermagnetic spin splitting the authors claim to observe. It is thus expected to substantially change the Fermi surface, possibly including Lifshitz transitions.

(ii) At least equally important, I would expect SOC to be able to lift the nodal-plane degeneracies, thereby changing all orbit outlines passing through. As these degeneracies are key to the authors' line of argument, they must carefully assess the effects of SOC and, depending on the gap size, magnetic breakdown.
(iii) Experimental results like ARPES, Magnetization, and specific heat suggest that electronic correlations do play a role in CrSb and may be incorporated into the DFT calculations, e.g., in the DFT+U scheme, again changing the Fermi surface from the plain vanilla case. The authors should comment on that.
Given points (i)-(iii), it is important to note that the authors have to apply a large ad hoc shift of the Fermi level of more than 100meV, inducing an ad hoc Lifshitz transition, in order to assign a specific orbit to a closed "dog's bone" pocket that is actually an open sheet in their own calculations and in experimental ARPES results.
While ad-hoc shifts are a well-established way of adjusting minor quantitative differences between the experimental facts and naturally imperfect DFT results, I am not inclined to accept such an ad hoc change of the Fermi surface topology without having seen the effects of SOC and DFT+U first, and without a thorough assignment of all orbits to all Fermi surface sheets.
From the perspective of assigning the dHvA orbits to the Fermi surface, the authors probably need to consider further (i) magnetic breakdown, assuming that the nodal planes are gapped in reality, and possibly (ii) the Zeeman effect on the already spin-split bands in the strong magnetic fields they use.

To summarize, the demonstration of altermagnetic g-wave symmetry first requires a full experimental determination of the Fermi surface, with a clear assignment of all observed orbits to all parts of the Fermi surface, definitely including SOC and possibly including correlations. After having convinced the reader that we know exactly from experiment how the Fermi surface looks and which of the observed dHvA frequencies belong to which k-space orbit, the authors can single out specific dHvA branches whose angular behavior demonstrates the spin splitting, again including SOC and possibly magnetic breakdown. I would like to point out that this is also mandatory, because the measurements themselves are not spin-resolved. Instead, the demonstration via quantum oscillations relies on the differences between Fermi-surface shapes calculated for the altermagnetic and the non-altermagnetic case.

Some comparatively minor comments:
- The Extended Figures seem to be referenced backwards in the text, starting with Figure 8.
- Caption Fig. 1. A screw rotation is not a point group operation. Why is a dogs-bone shape an ellipsoid?
- Fig. 3a,b: Why are there different angles on the x-axes when they are for comparison?
- Explaining the strong amplitude differences between the rather similar but split orbits in Fig.3b and 4b might be beneficial for the reader.
- Duplicating data in the heatmaps of Fig.2 and Fig.3 prompts immediate doubts on the soundness of the results. Although the authors clarify what they did in the supplementary material, I suggest giving the careful reader an advance warning in the captions.

Referee #3

(Remarks to the Author)
In this work, the authors present a three-dimensional mapping of the g-wave magnetic order in the metallic altermagnet CrSb, achieved through direction-resolved quantum oscillation (QO) measurements. By rotating the magnetic field, they demonstrate that the QO patterns exhibit a single frequency when the field lies within the nodal plane, and frequency splitting when the field is oriented out of the plane. The observed features are in good agreement with symmetry analysis of the g-wave magnetic order and first-principles band structure calculations. Given the growing interest in altermagnetism, this work is timely and represents a significant contribution, with high-quality experimental measurements, theoretical analysis, and presentation. However, in its current form, I do not consider the manuscript suitable for publication in Nature. The following points should be addressed before I can provide a positive recommendation.

Main Concern: Indirect Evidence of Altermagnetism
1. My primary concern is that while the work discusses altermagnetic physics, the experimental results provide only indirect signatures of it. Specifically, although the altermagnetic symmetry is revealed, no direct signature associated with magnetism or electron spin is presented. In this respect, although this study employs QO—a different technique from the ARPES used in a recent work [Phys. Rev. Lett. 133, 206401 (2024)]—both studies provide evidence for altermagnetic spin structures only in a spin-integrated manner, relying on comparison with DFT calculations. The PRL paper should be cited, as it investigates the same material system using a complementary method.

Suggestion for Spin-Sensitive Analysis
2. Related to the above point, I would like to ask whether the authors could perform spin-resolved measurements or try to explore the spin information in their results. A straightforward way to access spin-related information would be to examine the effect of Zeeman splitting. The Zeeman field can couple opposite spin states along the nodal plane and may produce observable physical effects, such as the frequency splitting proposed in a recent theoretical work [Phys. Rev. B 111, 125119 (2025)]. The most interesting regime occurs when θ is near $\pi/2$, where the Zeeman field is nearly perpendicular to the Néel vector and may play an important role in coupling opposite-spin bands. The authors are encouraged to analyze the possible role of the Zeeman field. If its effect is negligibly small, a justification should be provided.

Other Comments:
3. The authors state: "Unconventional superconductors are those in which the momentum-dependent superconducting energy gap changes sign." This definition is too narrow. The term "unconventional superconductivity" refers broadly to superconductivity that cannot be described by the BCS theory, not solely to the sign-changing gap structure.

4. Similarly, the phrasing "Newly-classified altermagnets are collinear and magnetically compensated like antiferromagnets" is misleading, as it implies that altermagnets and antiferromagnets are two parallel or separate classes. In fact, altermagnets are a specific subtype of antiferromagnets. Other statements implying this distinction should be revised accordingly.

5. Typo: The sentence "nodal planes every θ=60 for rotations in the a−ab plane, and every φ=90 when rotating in the c−ab plane" appears to have θ and φ swapped. Please check and correct.

6. In Fig. 2a, a color bar should be included for clarity.

7. The Fermi surface of the system has a complex dogbone shape. On page 14, the authors discuss the effective masses of the material. It would be helpful to clarify whether such a simplified description in terms of effective mass is physically meaningful, given the complexity of the Fermi surface.

8. The prospect that "the current system opens numerous avenues for next-generation spintronic device applications" seems overstated. The concrete application potential remains unclear, and more importantly, the presence of additional Fermi pockets beyond the altermagnetic one further complicates the pathway toward practical device implementation.

9. The authors should clarify how the extremal cross-sections are obtained. Given the complexity of the Fermi surface, it is important to consider whether multiple extremal cross-sections may exist. If so, the minimal and maximal areas would play distinct roles, and typically, it is the minimal cross-sectional area that determines the QO frequencies.

Version 1:

Reviewer comments:

Referee #1

(Remarks to the Author)
I have carefully read the revised manuscript by Mengmeng Long et al, and found that the authors had modified the manuscript according to all the comments of three reviewers one by one. I agree to recommend the revised manuscript to be published in Nature.

Referee #2

(Remarks to the Author)
Mengmeng Long and colleagues addressed most of the concerns raised, not all to my satisfaction.
Most critically, it was not clear from the original manuscript whether the very few orbits on which the entire discussion focused were ambiguously assigned to the FS.

In particular, because the authors had to propose an ad-hoc energy shift to their calculations large enough to change the connectivity of the sheet under investigation from open to closed while at the same time neglecting the effects of SOC and correlations, this cast severe doubt on the reliability of the results – Only because something seems consistent with expectations, it is not strong evidence for correctness, as the authors point out themselves in their introduction for the case of recent ARPES results on altermagnetic candidates.

There was and still is no clear evidence presented why the orbits under consideration could not arise from other parts of the FS, when performing similar band shift / SOC / +U maneuvers.
I maintain that the canonical and arguably best way to achieve an unambiguous assignment of an orbit is by an unambiguous assignment of all orbits. Given the rather clear branches in their experimental spectra, I am surprised that the authors were unable to do this.

Alternatively, showing that the other sheets robustly cannot produce similar dHvA branches, regardless of band shifts, +U, and so on, within a reasonable range, would be the second-best option. Merely showing the consistency of the two assigned orbits with one sheet is weak. Simple example: If your calculations show a spherical pocket and you observe a dispersionless branch with a similar frequency, can you assign it? – Only when you are sure that there is no second spherical pocket with a similar frequency somewhere else within your range of calculational uncertainty.
The revised manuscript is still not entirely convincing in this regard. However, the DFT+SOC+U calculations are significant additions. +U could be identified as being most probably responsible for the Lifshitz transition, and SOC could be shown to (of course) alter the sheet connectivity, but with breakdown fields well below the experimental field range where QOs occurred.

The mass scaling of the two “focus” orbits is another nice piece of evidence, somewhat mitigating the missing information on the other orbits and sheets, because it represents a rather specific property requiring significant fine tuning for an accidental match. – On the other hand, it is a specific property of spin up/down sheet pairs, and not of the dogbone sheet pair as such. It could, in principle, also be due to a spin pair of other parts of the FS. After all, all sheets are altermagnetic pairs, as the authors point out.

To summarize that, I would like to see more specific statements/plots in the supplements clarifying that the other FS sheets

are unable to produce similar branches regardless of shift, SOC, and +U.
Another point worth improving is that the main body of the manuscript is basically still written, as if SOC and correlations did not exist. I find this irritating and in parts also misleading: A real material always has SOC, so I suggest starting with that and then arguing (referring to the supplements) why the data interpretation works without it (field-assisted tunnelling, inversion-symmetric crystal structure) , and not the other way around.

The following minor points remain to be addressed:
- Isotropic DeltaE does not translate to isotropic Deltak, except for isotropic dispersions E(k). I suggest rephrasing that.
- The SOC-orbits within a nodal plane (Fig S7 c): Why is that splitting not visible as branch splitting? Probably, the argument is that there is magnetic breakdown also in this scenario, which happens only when the splitting is not isotropic (see point above)?
- From first round: What was meant with "duplications" in the heat maps of the old Figures 2 and 3 is that the authors copied experimental spectra from below 90° to the symmetry-related positions above 90° without saying that in the caption or the main text. They mentioned this only in the extended data Figs. 4 and 5. I suggested to put a disclaimer in the main text or caption, because the inclined reader sees the duplicated noise patterns, and immediately wonders if the authors want to sell him data they did not measure – something I suggested to avoid right from the start.
More as a remark I would like to point the authors to [Nature 594, 374 (2021), PRL 129, 026401 (2022), Nat. Phys. 18 813 (2022)], where QO signatures of exact nodal planes (not related to altermagnetism) and their lifting are discussed. However, I am not sure that this should be incorporated into the present text, because it might cause more confusion than it resolves.

Referee #3

(Remarks to the Author)
I have read the authors' response and the targeted revisions to the manuscript. It must be said that the revised version is a great improvement over the first submission, including a careful discussion of the role of SOC, Lifshitz transitions of the Fermi surface, the possibility of magnetic breakdown, and the analysis of effective masses. Although this is a beautiful and important piece of work, there is no direct spin-related effect — all the results are supportive rather than definitive. I recommend publishing the paper in Nature Physics.

Version 2:

Reviewer comments:

Referee #2

(Remarks to the Author)
I consider the amendments made in response to the raised points sufficient.

Open Access This Peer Review File is licensed under a Creative Commons Attribution 4.0 International License, which

permits use, sharing, adaptation, distribution and reproduction in any medium or format, as long as you give appropriate credit to the original author(s) and the source, provide a link to the Creative Commons license, and indicate if changes were made.
In cases where reviewers are anonymous, credit should be given to 'Anonymous Referee' and the source.
The images or other third party material in this Peer Review File are included in the article's Creative Commons license, unless indicated otherwise in a credit line to the material. If material is not included in the article's Creative Commons license and your intended use is not permitted by statutory regulation or exceeds the permitted use, you will need to obtain permission directly from the copyright holder.
To view a copy of this license, visit https://creativecommons.org/licenses/by/4.0/

Referee #1 (Remarks to the Author):

Recently, there have been many reports on the electronic band, magnetic structure, electronic transport properties of CrSb, which is the typical example of altermagnets, as a novel, third class of magnet, alongside with ferro- and antiferromagnets. Especially, the occurring of a spin-splitting electronic bands caused by the atomic exchange interaction in this material has attracted much attention. In this manuscript, Mengmeng Long et al. confirmed that this altermagnetic band structure leads to the loss of mirror symmetry for each spin-split Fermi sheet away from highly symmetric nodal orientations; the difference between up and down spins follows the profile of the spherical harmonic, analogous to a g-orbital of the H atom, by using the magnetic quantum oscillation (QO) measurements with different orientations of magnetic field relative to the low and high-symmetry directions of its Brillouin zone. These results are interesting for understanding details of the spin-splitting in the metallic altermagnets, and the experimental data are reliable. However, whether the reported results, or the used method (the QO measurements by means of cantilever at high magnetic field), or the studied material are not so new. I think that the manuscript is inadequate to be published in Nature, while I agree to recommend it to be published in Nature Phys. or Nature Commun.

We thank the referee for their positive assessment of the reliability of our results. We of course have a difference of opinion regarding the impact and novelty of the results. We would politely argue that: (i) finding a metallic altermagnet is highly desirable for spintronic memory applications; (ii) early promising candidates like RuO_2 and KV_2Se_2O have proved **not** to be bulk altermagnets; (iii) our study is the first to demonstrate intrinsic altermagnetic ordering *throughout the bulk* of a material; (iv) in addition to the mirror symmetry arguments of our rotational study, another key piece of corroborating evidence comes from the scaling between QO frequency and effective mass. This is a property that ARPES cannot probe, and gives <u>perfect agreement</u> between theory and experiment for the spin-split Fermi surface sheets we resolve in this study. In our revised manuscript we now do more to highlight this important aspect of our dataset, which we believe is a novel 'smoking gun' fingerprint of altermagnetism not previously considered, which goes significantly beyond prior studies.

Referee #2 (Remarks to the Author):

Mengmeng Long and colleagues report the growth and characterization of high-quality single-crystalline CrSb and the investigation of its Fermi surface properties by quantum oscillation experiments with the goal of demonstrating the predicted altermagnetic g-wave symmetry of the spin splitting experimentally.
The topic is of great current interest, and a conclusive demonstration of the altermagnetic spin splitting in a good metal would be an important step forward. However, results like these being awaited by the community may have created some pressure towards early submission.

The paper is written in clear language, and the work's significance is well presented. The experimental data seem well-suited for the task at hand.
However, the manuscript has severe shortcomings in its current form, so there is no unambiguous demonstration of the altermagnetic g-wave spin splitting.
The main problems lie at the heart of the quantum oscillatory study: to demonstrate the

unconventional g-wave spin structure, a complete Fermi surface determination is mandatory, because otherwise it remains unclear if the specific orbits the authors singled out for their case are really the ones they assume they are. However, this has not been done at all. The experimental data may well be sufficient for the task; however, we cannot tell because no attempt is made to assign all orbits to the Fermi surface.

In this resubmission, we now include 21 pages of *Supplementary Information*, which includes an extensive discussion of our calculational parameters and considerations of the Fermi surface of CrSb. We have also made significant changes to the introduction of the paper, to streamline the key concepts underpinning this work.

We respectfully disagree with the referee in their assertion that in order to identify altermagnetic spin-splitting (in this case, of *g*-wave character) we must necessarily "…assign all orbits to the Fermi surface." Instead, we would argue that so long as **one** orbit could be conclusively identified, and shown to possess the same symmetry properties in its spin-split rotational profile as that of a hydrogenic *g*-orbital, this would itself give strong evidence in favour of *g*-wave magnetic order parameter symmetry. Now, of course, all other Fermi sheets must necessarily also possess this symmetry. However, the utility of identifying the <u>first</u> singular orbit represents ~100% of the total value in this exercise – the identification of all the subsequent orbits/bands provides only small marginal value, we would argue, once that first conclusive determination has been made.

Of course, it would be preferable, instead, to have a full and clear determination of the Fermi surface, and as the referee comments, to unambiguously link all orbits with specific, distinct Fermi sheets. While on the one hand the universe seems to have been generous in conspiring to provide to us in CrSb, relatively early in the field of altermagnetism, an archetypical metallic example with a very high ordering temperature and clean specimens suitable for numerous technological applications – on the other hand, for the purposes of this specific task of going through orbit by orbit, sheet by sheet, things are much less generous. The outer web sheet of CrSb is very complicated, with several extremal orbit areas that produce a complicated spaghetti-like mass of frequencies. This is why we focus predominantly on the central 'dogbone' Fermi pocket, where things are much cleaner and clearer in the frequency range above that of the web.

We note that, we believe we are very well justified in our assignment of high frequency (~3-4kT) orbits to the central dogbone DFT pocket due to the remarkably good correspondence between the square root of the ratio of frequencies with the ratio of effective masses (main Fig. 4). This is exactly as expected for two spin-split Fermi sheets of the same mother pocket, due to the frequency scaling quadratically in the Fermi wavevector, whereas the mass is linearly proportional. We have reworded sections of the revised manuscript to better highlight this excellent correspondence, which we believe is a key fingerprint signature of quantum oscillations in an altermagnet that has not previously been considered. Due to the very good agreement (to two significant figures) between these two ratios, this corroboration gives us very good confidence that we have correctly identified these two frequency components as coming from the spin-split sisters of the same mother pocket, which from DFT calculations can only be the large central (dogbone) pocket. In our revised manuscript we now give an extended mass analysis, which demonstrates that these ~3-4kT Fourier components cannot be from breakdown/harmonics, but clearly correspond to fundamental frequency components. For these numerous reasons, we feel well justified in our determination of which orbits stem from

the central dogbone pocket, which clearly exhibit the hallmark signatures expected for a spin-split metallic altermagnet.

The presented DFT calculation for the Fermi surface is insufficient:
(i) Most severely, the authors completely neglect spin-orbit coupling (SOC) in their calculations and discussions. I do not see any justification for that: SOC is expected to alter the band splittings on an energy scale in the same ballpark as the altermagnetic spin splitting the authors claim to observe. It is thus expected to substantially change the Fermi surface, possibly including Lifshitz transitions.
(ii) At least equally important, I would expect SOC to be able to lift the nodal-plane degeneracies, thereby changing all orbit outlines passing through. As these degeneracies are key to the authors' line of argument, they must carefully assess the effects of SOC and, depending on the gap size, magnetic breakdown.
(iii) Experimental results like ARPES, Magnetization, and specific heat suggest that electronic correlations do play a role in CrSb and may be incorporated into the DFT calculations, e.g., in the DFT+U scheme, again changing the Fermi surface from the plain vanilla case. The authors should comment on that.

The referee raises an excellent point about the role of spin orbit coupling. This has motivated us to perform much more detailed calculations of the effects of SOC, and the associated impact on breakdown orbits. In our original submission we elected to try and keep the paper as simple as possible, only tangentially referring to such effects. However, the referee is quite right that we should give a full and thorough exposition of these points, which our new *Supplementary Information* goes into at length.

The key takeaway is that **these effects do not change our ability to deduce the *g*-wave altermagnetic order parameter symmetry**. This can be seen by considering the below figure:

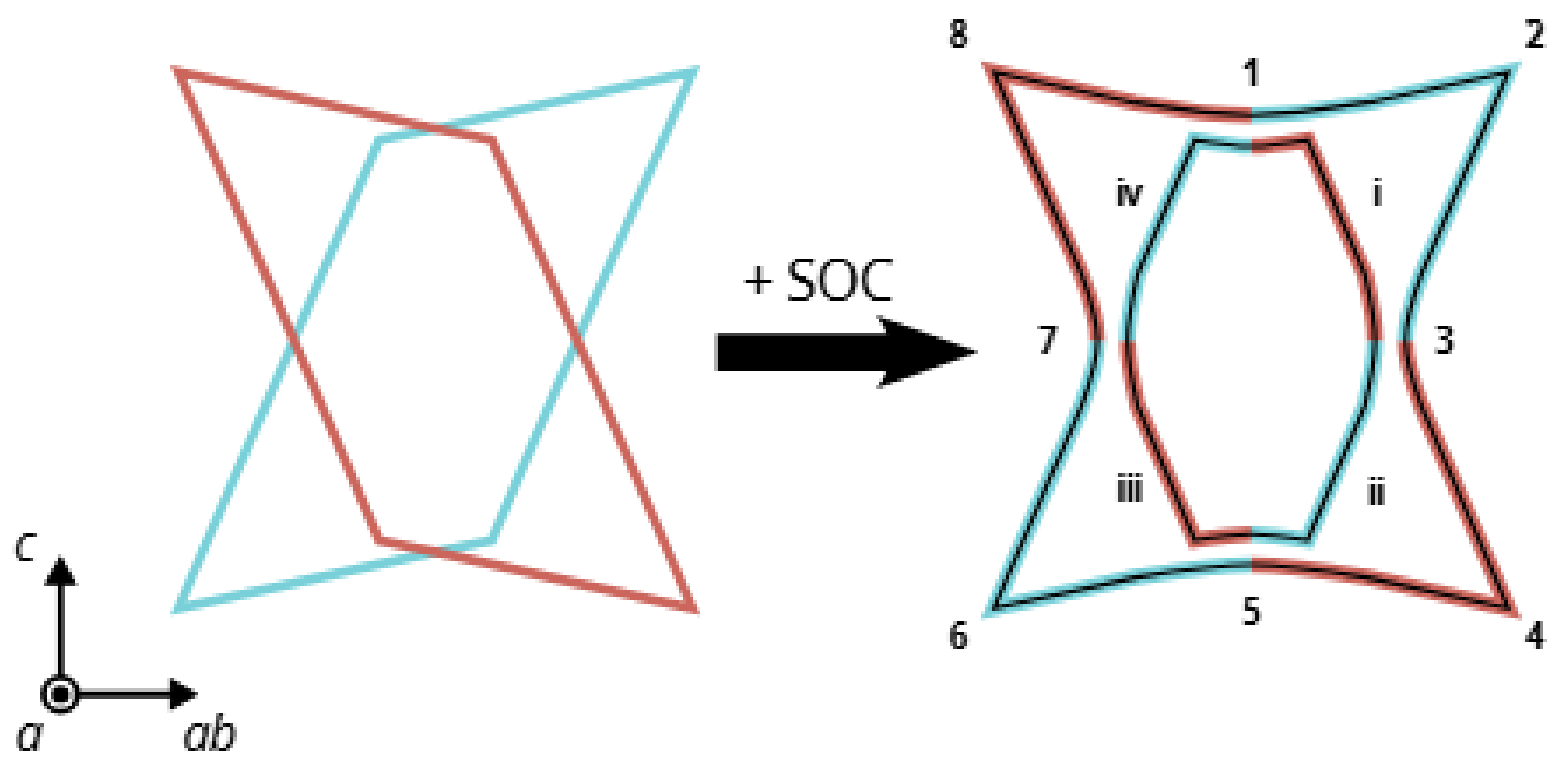

Fig. S5. Schematic of magnetic breakdown at SOC-induced avoided crossings. (Left) Pristine, unhybridised Fermi surface cross-sections as viewed down the *a*-axis. Along this direction, the cross-sections can be modelled as intersecting parallelograms, where spin-up (red) and spin-down (blue) sheets are mirror reflections of each other about the nodal planes. (Right) The introduction of SOC opens hybridisation gaps at intersection points. In high

magnetic fields, quasiparticles follow the pristine path (e.g., 1-i-3-ii-5-iii-7-iv-1) via magnetic breakdown tunnelling (points 1, 3, 5, 7) rather than the hybridised outer path (1-2-3-4-5-6-7-8-1).

While SOC may indeed introduce small *k*-space gaps between Fermi sheets, it does so at an *even* number of crossing points. Therefore, if a quasiparticle starts its orbit on the up (down) sheet, after hopping around an even number (in this case four) of crossings it will still return to the same original location on its up (down) sheet. Indeed, it **always** tunnels across to its same-spin flavour (i.e. always traces out a fully red or fully blue path). If there were an odd number of crossing points this would be an entirely different story, and QOs would not be able to discern the magnetic order parameter symmetry. However, as we go into great detail in the supplement to explain, for the case of CrSb this is not an issue.

We also consider the likelihood of significant SOC-induced Fermi surface reconstruction. However, we find this to be negligible, as also detailed in our new supplement:

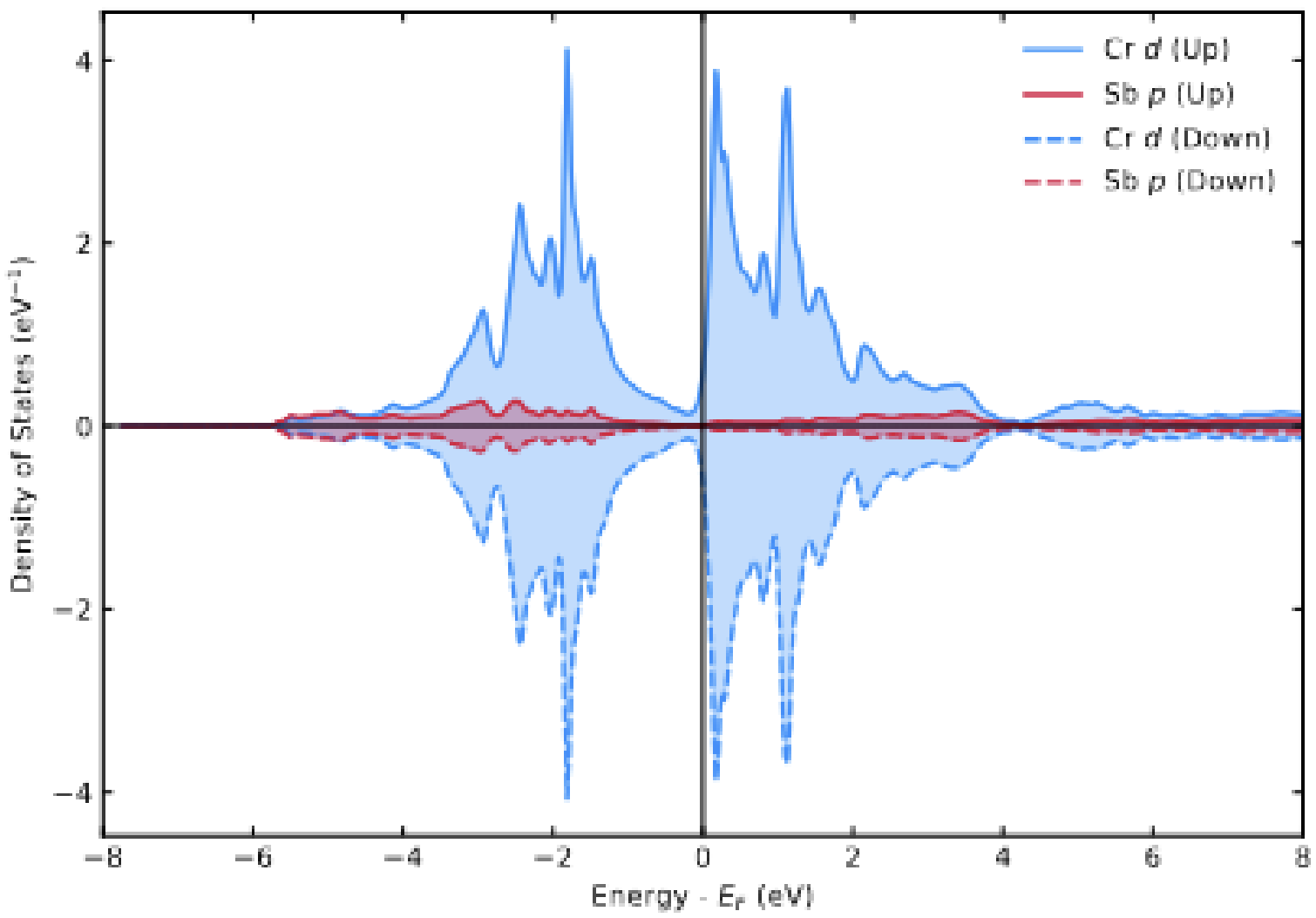

Fig. S3. Projected density of states (PDOS) onto the antimony *p*-orbitals and chromium *d*-orbitals of CrSb The electronic structure near the Fermi level (E_F = 0eV) is primarily dominated by chromium 3d-states (light blue), with a significantly smaller contribution from antimony 5p-orbitals (red). The spin-up (solid lines) and spin-down (dashed lines) channels are shown on the positive and negative axes, respectively. This dominance of light transition metal d-states suggests that the contribution of SOC at the Fermi surface should be small, inducing hybridisation but not substantial reconstruction.

We politely point the referee to our new supplement, which goes into much further detail regarding these excellent questions the referee has raised.

Given points (i)-(iii), it is important to note that the authors have to apply a large ad hoc shift of the Fermi level of more than 100meV, inducing an ad hoc Lifshitz transition, in order to assign a specific orbit to a closed "dog's bone" pocket that is actually an open sheet in their own calculations and in experimental ARPES results.

While ad-hoc shifts are a well-established way of adjusting minor quantitative differences between the experimental facts and naturally imperfect DFT results, I am not inclined to accept such an ad hoc change of the Fermi surface topology without having seen the effects of SOC and DFT+U first, and without a thorough assignment of all orbits to all Fermi surface sheets. From the perspective of assigning the dHvA orbits to the Fermi surface, the authors probably need to consider further (i) magnetic breakdown, assuming that the nodal planes are gapped in reality, and possibly (ii) the Zeeman effect on the already spin-split bands in the strong magnetic fields they use.

Regarding the Lifshitz transition, motivated and guided by the referee's comments, we have considered the effects of correlations on the Fermi surface geometry and topology. Here we reproduce Fig. S2, which shows how no ad hoc shift is required if one considers adding in the effects of electronic correlations through a Hubbard *U* of >2eV.

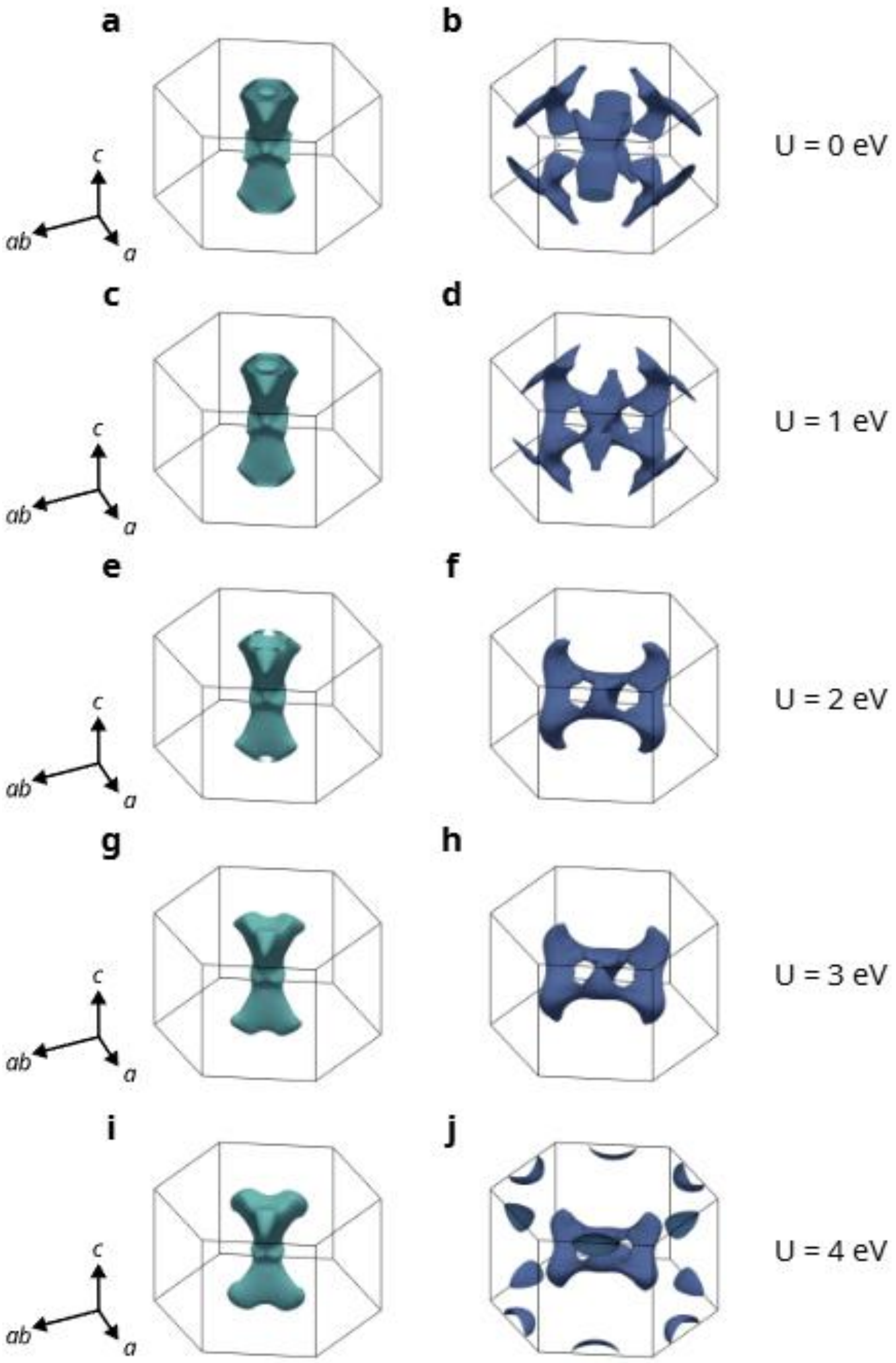

Fig. S2. Correlation-induced Lifshitz transition of the electron Fermi surface. (a,b) Standard DFT calculations (U=0eV) predict an open cylindrical topology for the central electron sheet (blue). Increasing the Hubbard interaction through (c,d) U=1eV and (e,f) U=2eV progressively pinches the sheet at the Brillouin zone boundaries. A Lifshitz transition occurs between 2 and 3eV, resulting in the formation of the closed, isolated 'dogbone' pocket observed at (g,h) U=3eV and (i,j) U=4eV, which matches the experimental observation.

To summarize, the demonstration of altermagnetic g-wave symmetry first requires a full experimental determination of the Fermi surface, with a clear assignment of all observed orbits to all parts of the Fermi surface, definitely including SOC and possibly including correlations. After having convinced the reader that we know exactly from experiment how the Fermi surface looks and which of the observed dHvA frequencies belong to which k-space orbit, the authors can single out specific dHvA branches whose angular behavior demonstrates the spin splitting, again including SOC and possibly magnetic breakdown. I would like to point out that this is also mandatory, because the measurements themselves are not spin-resolved. Instead, the demonstration via quantum oscillations relies on the differences between Fermi-surface shapes calculated for the altermagnetic and the non-altermagnetic case.

We thank the referee for their very thoughtful comments and suggestions for improvements. We hope that they find our new, very detailed, *Supplementary Information* to assuage their concerns regarding our theoretical considerations of the Fermi surface topology and geometry in CrSb.

Regarding the question of a lack of spin-resolution, in discussing our work with numerous colleagues over recent weeks, we regret that we did not make more effort, in our original draft, to highlight the remarkable effective mass/QO frequency scaling we observed (main Fig. 4). For the ratio of the spin-split cyclotron effective masses to be exactly equal to the square root of the ratio of the spin-split frequencies – to within 2 significant figures – we think, on reflection, is an exceptionally well-resolved empirical confirmation of altermagnetism in CrSb. To play devil's advocate for a moment, for the observed split QO frequency branches *not* to be due to altermagnetic spin-splitting, a sceptical reader might argue that the observation of their rotational profile fitting extremely well with the predicted rotational profile of altermagnetic nodal planes – and them being mirror-symmetric about those planes – is just some remarkable coincidence of unusual Fermi surface geometry. However, the fact that the sqrt ratio of frequencies *exactly* matches the ratio of masses – to such a high level of precision – would, we think, require some astronomically remote probability in this hypothetical case. We therefore believe that Occam's razor points overwhelmingly to our conclusion that CrSb is indeed a metallic, bulk, *g*-wave altermagnet.

Some comparatively minor comments:
- The Extended Figures seem to be referenced backwards in the text, starting with Figure 8.
- Caption Fig. 1. A screw rotation is not a point group operation. Why is a dogs-bone shape an ellipsoid?
- Fig. 3a,b: Why are there different angles on the x-axes when they are for comparison?
- Explaining the strong amplitude differences between the rather similar but split orbits in Fig.3b and 4b might be beneficial for the reader.

- Duplicating data in the heatmaps of Fig.2 and Fig.3 prompts immediate doubts on the soundness of the results. Although the authors clarify what they did in the supplementary material, I suggest giving the careful reader an advance warning in the captions.

We are not sure what 'duplication' the referee is referring to – no data in Fig. 2 is included/reproduced in Fig. 3. These are two independent datasets, of different rotational planes. We are also unsure about the 3b/4b point raised. We take this point to perhaps relate to the fact that, in Fig. 4b, the two QO amplitudes are quite different. This is very likely due to the curvature of the Fermi surface, which is clear in Fig3b that DFT predicts the up spin sheet to be quite sharp here – which then makes sense why the intensity at those sharp corners of the freq vs angle trace in Fig3a are at lower intensity compared to the much smoother evolution of the down spin sheet over this range, where the change in Fermi surface curvature is significantly more gradual. Regarding the phrasing of dogbone/ellipsoid, here we meant merely to politely inform the reader that this pocket is 3D in character – not, as asserted by numerous ARPES/DFT studies, an open quasi-2D cylinder. We have now made a small amendment so that this passage reads: "The central Fermi sheet of CrSb is a closed ellipsoid-like pocket of hole character, shaped like a dogbone". We hope that the referee finds this acceptable (it is, in effect, a deformed ellipsoid, we would argue). We have rectified the discussion of screw rotation, and now number the Extended Figures chronologically through the text.

We are very grateful to the referee for raising these numerous points regarding SOC, correlations and breakdown, which have motivated us to produce a detailed *Supplementary Information*. We feel that the supplement very strongly corroborates the finding of the main text – namely, that we understand the dogbone pocket of CrSb very well, and by tracking its angular profile through three distinct rotation planes, we have quite clearly shown that it exhibits the QO symmetry properties expected for altermagnetic spin splitting. Furthermore, the scaling between the ratios of frequencies and masses constitutes a fingerprint signature of a spin-split band structure, to our knowledge not previously theoretically considered, which here we empirically demonstrate is present in CrSb. We therefore feel our study provides a large body of evidence in favour of CrSb being the first empirically-confirmed material to exhibit bulk altermagnetic ordering. We thank the referee for their constructive comments in helping us to refine the presentation of our results, and given the significant changes and additions outlined above, we hope they feel our study is now ready for publication.

Referee #3 (Remarks to the Author):

In this work, the authors present a three-dimensional mapping of the g-wave magnetic order in the metallic altermagnet CrSb, achieved through direction-resolved quantum oscillation (QO) measurements. By rotating the magnetic field, they demonstrate that the QO patterns exhibit a single frequency when the field lies within the nodal plane, and frequency splitting when the field is oriented out of the plane. The observed features are in good agreement with symmetry analysis of the g-wave magnetic order and first-principles band structure calculations. Given the growing interest in altermagnetism, this work is timely and represents a significant contribution, with high-quality experimental measurements, theoretical analysis, and presentation. However, in its current form, I do not consider the manuscript suitable for publication in Nature. The following points should be addressed before I can provide a positive recommendation.

We thank the referee for their assessment of our “timely… significant contribution” with “high-quality experimental measurements”.

Main Concern: Indirect Evidence of Altermagnetism

1. My primary concern is that while the work discusses altermagnetic physics, the experimental results provide only indirect signatures of it. Specifically, although the altermagnetic symmetry is revealed, no direct signature associated with magnetism or electron spin is presented. In this respect, although this study employs QO—a different technique from the ARPES used in a recent work [Phys. Rev. Lett. 133, 206401 (2024)]—both studies provide evidence for altermagnetic spin structures only in a spin-integrated manner, relying on comparison with DFT calculations. The PRL paper should be cited, as it investigates the same material system using a complementary method.

The referee raises an excellent point. As we mentioned in our responses above, while we feel our original draft did a good job of conveying the geometrical interpretations of our study, we regret that we did not make more effort to highlight the remarkable effective mass/QO frequency scaling we observed (main Fig. 4). For the ratio of the spin-split cyclotron effective masses to be *exactly* equal to the square root of the ratio of the spin-split frequencies (to a precision of two significant figures) is, we feel, an exceptionally well-resolved empirical confirmation of altermagnetism in CrSb. This stems from very simple arguments relating the frequency of QOs to the enclosed Fermi surface cross-sectional area and hence scales quadratically with Fermi wavector, while all mass renormalisation factors cancel except for the difference in orbit circumferences that scales linearly in Fermi wavevector. **Not only has this expectation not previously been made from a theoretical viewpoint, here we empirically confirm it**. In so doing, we believe this constitutes a novel, high confidence smoking-gun signature of altermagnetism – far more robust than e.g. comparison of ARPES spectra with DFT band structures. Regarding the referee’s point concerning the previous body of work, we have restructured our introduction and edited the references, which we hope the referee now finds to give a better overview of recent work in this area.

Suggestion for Spin-Sensitive Analysis

2. Related to the above point, I would like to ask whether the authors could perform spin-resolved measurements or try to explore the spin information in their results. A straightforward way to access spin-related information would be to examine the effect of Zeeman splitting. The Zeeman field can couple opposite spin states along the nodal plane and may produce observable physical effects, such as the frequency splitting proposed in a recent theoretical work [Phys. Rev. B 111, 125119 (2025)]. The most interesting regime occurs when θ is near π/2, where the Zeeman field is nearly perpendicular to the Néel vector and may play an important role in coupling opposite-spin bands. The authors are encouraged to analyze the possible role of the Zeeman field. If its effect is negligibly small, a justification should be provided.

We thank the referee for raising this very interesting point. Unfortunately, we do not observe the predicted frequency splitting effects. We performed pulsed field measurements presented in Extended Data Fig. X, up to 64T, and cannot resolve any spin-flop/flip signatures, nor Zeeman effects, over the magnetic field range of our measurements. We suppose this is likely due to the very high ordering temperature of CrSb, pushing such effects to unaccessibly high field scales >100T. We have added a justification of this as the referee advises.

We would politely note that our observed scaling between the ratio of the square root of f1 and f2 with the ratio of m*1 and m*2 (Fig. 4), which agrees to a precision of two significant figures, is itself a spin-sensitive result. This result comes from very simple arguments relating the scaling of Fermi wavevector to go quadratically with Fermi surface area (and hence QO frequency), yet linearly with the effective cyclotron mass. Thus, while our angular-dependent study provides the first empirical evidence for bulk altermagnetic ordering in a material, we feel this additional aspect of the data provides a novel smoking-gun confirmation, which is theoretically expected in the case of spin-splitting, yet highly unlikely to just be some coincidence given the accuracy and precision with which we determined the frequencies and masses. We therefore feel our study is doubly impactful – first we show the expected geometrical altermagnetic properties in bulk-sensitive QO measurements, then we provide this additional novel scaling result that serves as a unique fingerprint of metallic altermagnetism.

Other Comments:
3. The authors state: "Unconventional superconductors are those in which the momentum-dependent superconducting energy gap changes sign." This definition is too narrow. The term "unconventional superconductivity" refers broadly to superconductivity that cannot be described by the BCS theory, not solely to the sign-changing gap structure.

We have amended our description of this to the following passage; "Unconventional superconductors are those where the pairing symmetry breaks crystal point group symmetries, or where the gap function exhibits a non-trivial phase structure (like a sign change) not required by the crystal symmetry." We hope that the referee finds this more nuanced phrasing to be an acceptable description, suitable for comparing the similarities between the very established field of unconventional superconductivity with the nascent developing one of unconventional magnetism, wherein it is the concept of 'nodal' points (or planes), and their associated symmetry properties, that is central to our present work.

4. Similarly, the phrasing "Newly-classified altermagnets are collinear and magnetically compensated like antiferromagnets" is misleading, as it implies that altermagnets and antiferromagnets are two parallel or separate classes. In fact, altermagnets are a specific subtype of antiferromagnets. Other statements implying this distinction should be revised accordingly.

We thank the reviewer for this comment regarding the classification of altermagnets. We recognise that under traditional nomenclature, any collinear magnetically compensated system is often categorised as an antiferromagnet. However, our phrasing follows the emerging symmetry-based classification established in recent seminal works (e.g., Šmejkal et al., Phys. Rev. X 12, 031042). Here, altermagnets are distinguished as a distinct third class of magnetism because their spin-split electronic structure is protected by non-relativistic rotational symmetries rather than the PT or tT symmetries characteristic of conventional antiferromagnets. To ensure clarity and avoid confusion for readers accustomed to the broader definition, we have revised the manuscript to acknowledge that while altermagnets share the property of being magnetically compensated with antiferromagnets, they are distinct in these symmetry properties. We believe that these distinct spin group symmetry properties justify this description, and that this emerging understanding is starting to be adopted across the community.

5. Typo: The sentence "nodal planes every θ=60 for rotations in the a−ab plane, and every ϕ=90 when rotating in the c−ab plane" appears to have θ and ϕ swapped. Please check and correct.

This is indeed a silly typo, for which we are very grateful to the referee for catching. This has now been amended.

6. In Fig. 2a, a color bar should be included for clarity.

Having considered this suggestion, we found including it in the figure is quite squashed, so we have added a line to the caption that explains that brighter heatmap intensities correspond to larger quantum oscillatory amplitudes. We hope this is now unambiguously clear to the reader.

7. The Fermi surface of the system has a complex dogbone shape. On page 14, the authors discuss the effective masses of the material. It would be helpful to clarify whether such a simplified description in terms of effective mass is physically meaningful, given the complexity of the Fermi surface.

Yes, the inverse relationship between Fermi velocity v_F and effective mass m* is fundamental. Moreover, as mentioned above, in discussions with theory colleagues, we have realised that the strong correspondence between the sqrt ratio of frequencies with the ratio of masses, for the spin-split QO components, is an important smoking-gun altermagnetic signature of our dataset. In our revised draft we have done more to bring out this important feature of our study. Furthermore, in our revised manuscript we now present additional Lifshitz-Kosevich fits to the lower frequency components, demonstrating that the 3-4kT peaks we focus on cannot be explained by breakdown/as second harmonics, adding strong corroboration to our inferences regarding the dogbone structure of the central Fermi pocket of CrSb.

8. The prospect that "the current system opens numerous avenues for next-generation spintronic device applications" seems overstated. The concrete application potential remains unclear, and more importantly, the presence of additional Fermi pockets beyond the altermagnetic one further complicates the pathway toward practical device implementation.

We would politely note that **all** Fermi sheets are spin-split in a metallic altermagnet. This stands for the web (electron pockets) as well as the dogbone (hole pocket). We chose to focus our analysis on the dogbone, as it is easier to evaluate which orbits correspond to which sections of the Fermi surface, and by high-pass filtering the data we can exclude the spaghetti-like mass of frequencies from the web and clearly evaluate the spin-split structure of the dogbone.

Regarding the wording of potential applications of altermagnets, we have amended our introduction and conclusion. We now finish the paper by saying "In combination, these results thereby firmly establish CrSb as a room-temperature metallic altermagnet. Furthermore, our optimised growth procedure has produced high-quality crystals with residual resistivities as low as 2~$\upmu \Omega$cm. Given that CrSb is relatively insensitive to air and moisture, it can be synthesised to high purity, the altermagnetic ordering temperature is very high, and both chromium and antimony are relatively abundant easily-sourced elements, this combination of favourable properties positions CrSb as a leading material for pursuing applications in next-generation low-energy spintronic devices."

We hope the referee finds this to be suitably justified phrasing.

9. The authors should clarify how the extremal cross-sections are obtained. Given the complexity of the Fermi surface, it is important to consider whether multiple extremal cross-sections may exist. If so, the minimal and maximal areas would play distinct roles, and typically, it is the minimal cross-sectional area that determines the QO frequencies.

Quantum oscillatory frequency components in thermodynamic variables analytically related to the free energy directly correspond to extremal areas of the Fermi surface. This is true for both maximal and minimal cross-sectional areas. In our new supplement we display numerous variations of Fermi surface renderings, and plot the theoretically expected QO frequencies versus rotation angle. While the web (hole) sheets always produce a spaghetti of many (low) frequencies, the central electron pocket shows several distinct characteristics across different calculations. This motivated us to refine our Fermi surface simulations, informed by our experimental QO data, resulting in our main-text choice of dogbone Fermi pocket rendering, which gives a very clear exposition of the split spin-up and spin-down contributions for magnetic field rotated through arcs of low symmetry, while Kramers spin degeneracy is symmetry-enforced in a nodal plane.

In summary, with our rewritten introduction and entirely new supplement, alongside numerous minor tweaks and changes throughout, we hope the referee now finds our results ready for publication.

Referees' comments:

Referee #1 (Remarks to the Author):

I have carefully read the revised manuscript by Mengmeng Long et al, and found that the authors had modified the manuscript according to all the comments of three reviewers one by one. I agree to recommend the revised manuscript to be published in Nature.

We thank the referee for their thoughtful consideration of our work.

Referee #2 (Remarks to the Author):

Mengmeng Long and colleagues addressed most of the concerns raised, not all to my satisfaction.

Most critically, it was not clear from the original manuscript whether the very few orbits on which the entire discussion focused were ambiguously assigned to the FS.

In particular, because the authors had to propose an ad-hoc energy shift to their calculations large enough to change the connectivity of the sheet under investigation from open to closed while at the same time neglecting the effects of SOC and correlations, this cast severe doubt on the reliability of the results – Only because something seems consistent with expectations, it is not strong evidence for correctness, as the authors point out themselves in their introduction for the case of recent ARPES results on altermagnetic candidates.

There was and still is no clear evidence presented why the orbits under consideration could not arise from other parts of the FS, when performing similar band shift / SOC / +U maneuvers.

I maintain that the canonical and arguably best way to achieve an unambiguous assignment of an orbit is by an unambiguous assignment of all orbits. Given the rather clear branches in their experimental spectra, I am surprised that the authors were unable to do this.

Alternatively, showing that the other sheets robustly cannot produce similar dHvA branches, regardless of band shifts, +U, and so on, within a reasonable range, would be the second-best option. Merely showing the consistency of the two assigned orbits with one sheet is weak. Simple example: If your calculations show a spherical pocket and you observe a dispersionless branch with a similar frequency, can you assign it? – Only when you are sure that there is no second spherical pocket with a similar frequency somewhere else within your range of calculational uncertainty.

The revised manuscript is still not entirely convincing in this regard. However, the DFT+SOC+U calculations are significant additions. +U could be identified as being most probably responsible for the Lifshitz transition, and SOC could be shown to (of course) alter the sheet connectivity, but with breakdown fields well below the experimental field range where QOs occurred.

We thank the referee for their patience in elaborating on their comments from the first review stage. We regret that we were not able to answer all of the referee's comments satisfactorily in our first revision but hope that this may be remedied with our latest additions to the Supplement, outlined below.

As stated by the referee, a **full unambiguous designation of each measured quantum oscillation frequency** to specific sections of the DFT-generated Fermi surface would be ideal. Such detailed agreement is **very rare in practice.** With the presence of transition metals in more complex materials, the number of bands near the Fermi level goes up, and the Fermiology necessarily becomes more complicated: the shortcomings of DFT may result in the band edges of the individual bands being slightly incorrect, which immediately causes changes in the geometry of the Fermi surface pockets, possibly even giving rise to entirely different pockets. This can to some degree be compensated by shifting the band edges until good agreement is obtained with experimental data, constraining the band shifts to keep overall charge balance. Such approaches have been very commonly applied in the analysis of QO measurements, a prominent example being measurements in iron pnictides.

In the case of CrSb, the **web (electron) pocket has a very complicated geometry,** which is hard to pin down using QO data at present. In particular, for some orientations, several of the orbits traverse Fermi surface sections of large curvature, for which very small QO amplitudes would be expected. This makes it very tricky to follow which strands of the band structure 'spaghetti' are associated with what oscillation frequency.

The frequency splitting observed for the highest frequency branch in the QO spectrum and the disappearance of this splitting for fields within certain high symmetry nodal planes indicate that the associated orbits arise from a single parent sheet that is spin-split by the exchange interaction. The magnitude of the QO frequencies and their field angle dependence suggest that this is the dogbone sheet identified in our paper. It would not, however, materially affect our argument, if these split branches were assigned to another pocket of the Fermi surface. The principal result remains the same, namely that the angle dependence of this branch of the QO spectrum is caused by spin splitting with distinct nodal and antinodal planes. **The robust outcome of our study is the precise mapping of nodal and antinodal planes that govern the splitting of QO frequency branches.** The same nodal and antinodal splitting directions are expected to apply also to all the other branches of the QO spectrum, but they could be observed most clearly and unambiguously in the branch we associate with the dogbone sheet.

Moreover, we are certain that **the high frequency branch discussed above does indeed arise from the dogbone sheet:** firstly, this assignment is suggested straightforwardly by DFT calculations with only minimal tweaking; secondly, even if there were no DFT results, the measured angle dependence would suggest a pocket with such a shape. Thirdly, we have followed the referee's suggestion (the 'second-best option') to check whether the high frequency branch discussed above might by any stretch of the imagination be attributed to any other sheet of the Fermi surface than the dogbone (hole) pocket and found that this is not possible.

We reproduce here new supplementary figure S8, in which we label several extremal orbits about the web sheet:

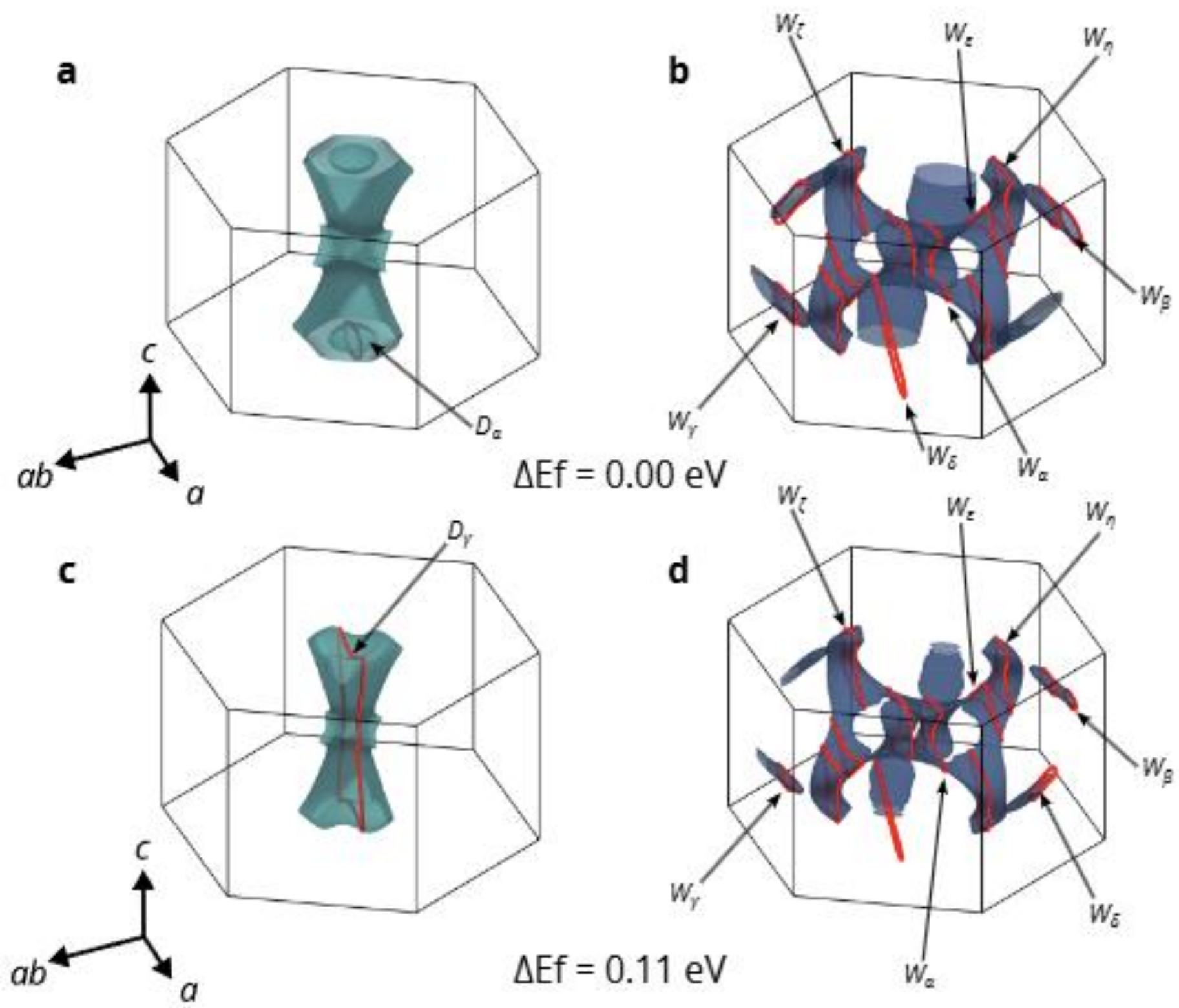

In our revised Supplement we now have a new Section 1.5, in which we carefully consider the possible cyclotron orbits that would be expected, for field oriented close to the *ab* direction, for a broad range of Fermi level shifts and Hubbard *U* values. Several of these yield expected QO vs angle plots that give very poor correspondence with the measured dHvA data – but as suggested by the referee, the aim of this exercise is to check whether the 'focus orbits' at 3-4kT in the main text might conceivably be assigned to any FS sheet other than the dogbone pocket.

In Section S1.5 we present comprehensive tables of SKEAF-deduced QO frequencies expected for these various web sheet orbits, **each of which are <2kT.** This rules out that the 3-4kT 'focus orbits' could arise from the web sheet.

The only way a higher orbit frequency of order 3-4 kT appears in simulations involving the web sheet is by including spin orbit coupling (SOC). Below we reproduce this orbit (LHS, from Fig. S10) and its simulated frequency vs angle profile (RHS, from Fig. S12). However, we can rule out that our measurements picked up such an orbit: (i) our SOC/magnetic breakdown arguments provided in the Supplement suggest that the experimental signature of such an SOC enabled orbit would be extremely weak at the fields used in our measurements, (ii) the the field angle dependence of QOs expected from this hypothetical orbit differs drastically from the observed QO data in Fig. 2 of the main text, both for fields near *c*, where the hypothetical orbit gives rise to large and strongly angle-dependent oscillations, and for fields perpendicular to *c,* where conversely the hypothetical orbit frequencies are flat with angle, whereas the observed frequencies change drastically with angle.

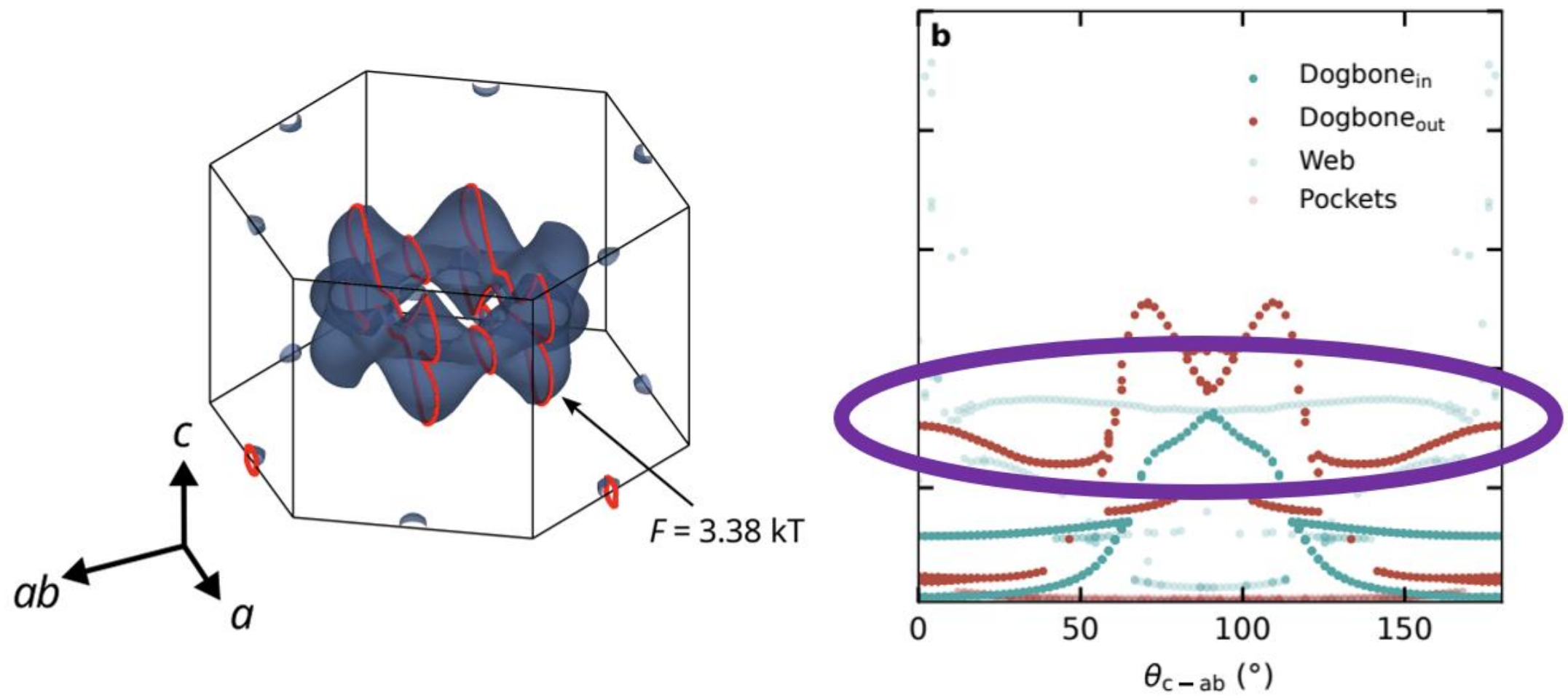

Note the flat form of the angle dependence of the simulated QO frequency for this orbit (faint blue circles in (b), highlighted with the purple ellipse). No such frequency branch is resolved in Main Fig. 2e. We therefore conclude that this hypothetical orbit does not contribute to our measured frequency vs angle QO spectra.

The new Section S1.5 in the Supplementary Information also explains why any frequency components in the 3-4 kT range cannot be attributed to higher harmonics of web sheet orbits. The observed effective masses should in such a case be multiples of the masses of the original fundamental orbits, which is contradicted by our mass studies. On similar grounds, they also cannot stem from magnetic breakdown between two distinct web sheet orbits, as this would also exhibit the sum of the two contributory effective masses, whereas all FFT peaks in Extended Data Fig. 9 show very similar temperature damping profiles, indicating they each correspond to fundamental QO frequencies.

In summary: comprehensive Fermi surface simulations for a broad range of parameters have not produced any QO frequencies > 2kT arising from the web sheet of the FS ("Web Frequency" entries in the Tables in Section S1.5 are all <2kT). By contrast, once the dogbone pocket undergoes its Lifshitz transition to produce closed orbits for field oriented along *ab*, each calculation iteration predicts an orbit >2kT to be present. This gives us strong confidence that our selected 'focus orbits' in the main text are indeed from the dogbone pocket. Whereas the QO spectrum from the web pocket is complex and sensitive to computational details, the branches arising from the dogbone are robust and well separated from the lower frequency branches arising from the web. By focussing on this 'quiet' dogbone frequency range, we can therefore isolate the antinodal spin-splitting in a clear manner.

This indeed was always the way in which we framed this paper – less of a traditional QO FS study, and more focussed on this central aspect of the data that can resolve the key aspect of altermagnetic spin-splitting clearly. As pointed out by the referee, it is important to have confidence in the assignment of our 'focus orbits'. We thank the referee for encouraging us to make this case properly, which we hope the referee now feels has been achieved in section S1.5.

The mass scaling of the two "focus" orbits is another nice piece of evidence, somewhat mitigating the missing information on the other orbits and sheets, because it represents a rather

specific property requiring significant fine tuning for an accidental match. – On the other hand, it is a specific property of spin up/down sheet pairs, and not of the dogbone sheet pair as such. It could, in principle, also be due to a spin pair of other parts of the FS. After all, all sheets are altermagnetic pairs, as the authors point out.

We thank the referee for the positive appraisal on this point – it would require a great deal of fine tuning for this result not to come from two altermagnetically spin-split Fermi surface sheets. As outlined above, an extensive parameter study in our Fermi surface simulations (many of which yield little resemblance to the experimental data), has not produced a cyclotron orbit in the observed frequency range and with approximately the observed field angle dependence that does not come from the dogbone sheet. We therefore conclude that it is overwhelmingly likely that the LK study in Fig. 4 comes from spin-up and spin-down sections of the dogbone pocket.

To summarize that, I would like to see more specific statements/plots in the supplements clarifying that the other FS sheets are unable to produce similar branches regardless of shift, SOC, and +U.

We thank the referee for this suggestion and hope that the referee finds the addition of supplementary section S1.5 helpful.

Another point worth improving is that the main body of the manuscript is basically still written, as if SOC and correlations did not exist. I find this irritating and in parts also misleading: A real material always has SOC, so I suggest starting with that and then arguing (referring to the supplements) why the data interpretation works without it (field-assisted tunnelling, inversion-symmetric crystal structure) , and not the other way around.

To address this important suggestion, we have added the following paragraph to the main text:

"Real materials always exhibit some spin-orbit coupling (SOC) and many-body electronic correlations, meaning a purely non-relativistic framework is only ever an idealisation. Nevertheless, our symmetry-based interpretation remains robust. Because CrSb possesses an inversion-symmetric crystal structure, the spatial symmetries protecting the orientation of the nodal planes remain intact. Furthermore, under the intense magnetic fields utilised in our experiments, field-assisted tunnelling (magnetic breakdown) allows quasiparticles to traverse small hybridisation gaps opened by weak SOC, effectively restoring the pristine altermagnetic trajectories. As detailed in the \textit{Supplementary Information} (in which we also explicitly account for electronic correlations) these considerations justify our simplified symmetry picture introduced here, yielding a direct mapping between the quantum oscillation frequency spectra and the underlying altermagnetic order parameter."

We hope the referee now finds this discussion to be more rigorous and better motivated.

The following minor points remain to be addressed:

- Isotropic DeltaE does not translate to isotropic Deltak, except for isotropic dispersions E(k). I suggest rephrasing that.

- The SOC-orbits within a nodal plane (Fig S7 c): Why is that splitting not visible as branch splitting? Probably, the argument is that there is magnetic breakdown also in this scenario, which happens only when the splitting is not isotropic (see point above)?

We thank the referee for raising these good points – we have rephrased to make it clear that here we mean the case of the dispersion should be symmetric with respect to the crystal symmetry. We have also rephrased our definition of an 'altermagnetic order parameter' in terms of E instead of k, so that this is also generically applicable for insulating altermagnets (in which there is no Fermi surface).

- From first round: What was meant with "duplications" in the heat maps of the old Figures 2 and 3 is that the authors copied experimental spectra from below 90° to the symmetry-related positions above 90° without saying that in the caption or the main text. They mentioned this only in the extended data Figs. 4 and 5. I suggested to put a disclaimer in the main text or caption, because the inclined reader sees the duplicated noise patterns, and immediately wonders if the authors want to sell him data they did not measure – something I suggested to avoid right from the start.

We apologise to the referee for our prior misunderstanding on this point, and we thank the referee for explaining it to us. That is an excellent point, and one we are sorry to have overlooked. We have now explicitly stated this is the captions of Figures 2 & 3.

More as a remark I would like to point the authors to [Nature 594, 374 (2021), PRL 129, 026401 (2022), Nat. Phys. 18 813 (2022)], where QO signatures of exact nodal planes (not related to altermagnetism) and their lifting are discussed. However, I am not sure that this should be incorporated into the present text, because it might cause more confusion than it resolves.

We thank the referee for bringing these works to our attention. We agree that alluding to such nodal planes in the main text may confuse more readers than it helps – however, we have added appropriate references in the SI for the enthusiastic reader (notably, the Nat. Phys. paper is very relevant to our discussion of very small breakdown fields, drawing interesting similitudes with our present work).

Referee #3 (Remarks to the Author):

I have read the authors' response and the targeted revisions to the manuscript. It must be said that the revised version is a great improvement over the first submission, including a careful discussion of the role of SOC, Lifshitz transitions of the Fermi surface, the possibility of magnetic breakdown, and the analysis of effective masses. Although this is a beautiful and important piece of work, there is no direct spin-related effect — all the results are supportive rather than definitive. I recommend publishing the paper in Nature Physics.

We thank the referee for their positive assessment of the changes incorporated in the last revision of the manuscript. We are grateful to the referee for their constructive advice in helping us to improve our manuscript.

Regarding the question of supportive vs definitive information, we maintain that our study provides clear, direct evidence that CrSb is the first robustly-identified metallic altermagnet. A key benefit of QOs compared to experimental techniques such as ARPES is that we have the

ability to tilt the magnetic field through different rotation planes. So, while both ARPES and QOs can deduce the band structure at the Fermi level – and identify sections of the band structure where altermagnetic spin-splitting is theoretically predicted, which can be compared with data – QOs have the advantage of comparing for fields in antinodal planes – where the splitting is maximal – and nodal planes – where the splitting disappears. As Referee #2 pointed out above: “only because something seems consistent with expectations, it is not strong evidence for correctness”. QOs’ ability to robustly demonstrate the **absence** of spin-splitting in nodal planes acts as a crucial sanity check, raising the confidence in diagnosing altermagnetism in a candidate material.

Moreover, our measurements have the significant benefit of probing a bulk thermodynamic variable, making our results immune from any extrinsic surface effects. Further key evidence comes from the observed mass scaling between altermagnetically spin-split Fermi sheets (in Fig. 4). QOs are one of the most direct probes of effective mass in the solid state. As Referee #2 comments, this feature of our study represents a “specific property requiring significant fine tuning for an accidental match”. The excellent correspondence we observe goes far beyond any aspect of previous studies attempting to identify altermagnetism in a candidate material. This, we argue, brings our results far closer to being ‘definitive’ rather than just ‘supportive’.

Given the controversy surrounding first RuO_2 and more recently KV_2Se_2O, the robust empirical demonstration of a metallic altermagnet is sorely outstanding. We believe our work provides this – and not just that, but it so happens that CrSb has a number of highly desirable properties (outlined in the final sentence of our manuscript), further enhancing the impact of our work.
